# Purification and Transcriptomic Characterization of Hypertrophied Hepatic Stellate Cells From CDAHFD Mouse Liver

**DOI:** 10.1096/fj.202502655RR

**Published:** 2026-04-23

**Authors:** Marion Heckmann, Nour‐El‐Houda Djerir, Keola Greliche, Pierre‐Henri Commere, Julien Fernandes, Guillaume Sarrabayrouse, Bernard Hainque, Pascal Bigey, Virginie Escriou, Céline Hoffmann

**Affiliations:** ^1^ Université Paris Cité, CNRS, INSERM, UTCBS Paris France; ^2^ Cytometry Platform Institut Pasteur, Université Paris Cité Paris France; ^3^ Institut Pasteur, C2RT, UTechS Photonic BioImaging Paris France

**Keywords:** hepatic fibrosis, hepatic stellate cell, murine model, retinoid, transcriptomic

## Abstract

Hepatic stellate cells (HSC) are known for their major role in hepatic fibrosis. It is well established that upon liver injury, they undergo a transition from a quiescent state to an activated state and transdifferentiate into proliferative, fibrogenic myofibroblasts. Recently, it has been shown that different subpopulations of HSC co‐exist during fibrogenesis and play different roles in the establishment of fibrosis. We previously highlighted, in murine model and human biopsies, a specific subpopulation of hypertrophied HSC (hypHSC) which exhibits exacerbated retinoid droplets and were closely associated with collagen fibers. The present study focuses on the molecular characterization of hypHSCs isolated from a murine model of metabolic liver fibrosis thanks to an experimental strategy we developed and described here. Liver dissociation followed by density gradient and fluorescence assisted cell sorting allowed us to obtain highly pure hypHSC preparations. Then, a transcriptomic analysis (bulk RNAseq) of hypHSCs versus quiescent HSCs purified from healthy mouse liver was performed. This showed that hypHSCs' molecular signature differs from HSC subtypes already described in the literature, with a “hybrid” profile involved in the regulation of the immune system, the remodeling of extracellular matrix and exhibiting a deregulation of lipid metabolism. Our study highlights that a phenotype‐to‐molecular approach can provide complementary elements to single‐cell molecular approaches and provides additional insights into the plasticity of hepatic stellate cells in a context of hepatic fibrosis.

## Graphical abstract

The purification protocol for hypertrophied hepatic stellate cells (hypHSCs) developed in this study, using fibrotic and steatotic mouse livers (CDAHFD model), made it possible to obtain a high‐purity hypHSC preparation for transcriptomic analysis. The hypHSC molecular signature corresponds to a hybrid profile with inflammatory, catalytic, and extracellular matrix remodeling activities.

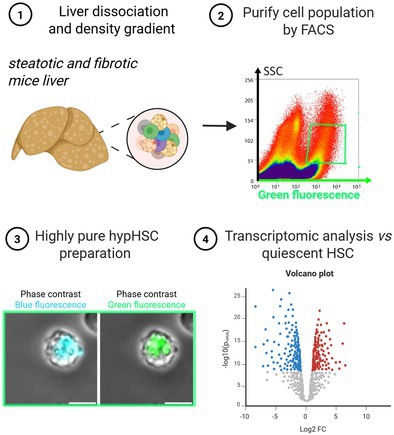

**Abbreviations**
αSMA: alpha smooth muscle actin
ASGPR: asialoglycoprotein receptor
BDL: bile duct ligation
CaHSCs: central vein‐associated HSCs
CCl_4_
: carbon tetrachloride
CD: cluster of differentiation
CDAHFD: choline deficient amino acid defined high fat diet
cDNA: complementary DNA
cRBP1: cellular retinol binding protein 1
DEGs: differentially expressed genes
DMEM: Dulbecco's modified eagle's medium
dNTP: deoxyribonucleoside triphosphate
EGTA: ethylene glycol‐bis‐(beta‐aminoethylether)‐N, N, N′, N′‐tetraacetic acid
FACS: fluorescence‐activated cell sorting
FBS: fetal bovine serum
FPKM: Fragments Per Kilobase Million
FSC: forward scatter
GBSS: Gey's balanced salt solution
GO: gene ontology
HEPES: acide 4‐(2‐hydroxyethyl)‐1‐piperazine ethane sulfonique
HF: high fat
HSC: hepatic stellate cell
hypHSC: hypertrophied hepatic stellate cell
IL7r: interleukin 7 receptor
LRAT: lecithin retinol acyltransferase
MAFLD: metabolic dysfunction‐associated fatty liver disease
MASH: metabolic dysfunction‐associated steatohepatitis
MCD: methionine and choline deficient
MMP: metalloproteinase
NASH: non‐alcoholic steatohepatitis
NGS: next generation sequencing
PaHSCs: portal vein‐associated HSCs
PAMP: pathogen‐associated molecular pattern
PBS: phosphate buffered saline
PDGFR‐β: platelet derived growth factor receptor beta
QC: quality control
qHSC: quiescent HSC
qPCR: quantitative polymerase chain reaction
RIN: RNA integrity number
SD: standard diet
sc‐RNAseq: single‐cell RNA‐seq
SSC: side scatter
TLR: toll‐like receptor
TNFα: tumor necrosis factor alpha
UV: ultraviolet

## 1 Introduction

Hepatic fibrosis, which corresponds to the excessive accumulation of extracellular matrix proteins in the liver parenchyma, is a major consequence of chronic liver diseases such as viral hepatitis, alcoholism, and metabolic dysfunction‐associated steatohepatitis (MASH, previously NASH) [1].

Hepatic fibrosis is a critical step in the course of the disease, as it can evolve into cirrhosis and hepatocellular carcinoma, which is one of the most serious cancers [2]. No specific treatment for hepatic fibrosis and its consequence is available; the only therapy is liver transplantation when the disease has reached its endpoints.

Among the liver cells, hepatic stellate cells (HSCs) are the major cells responsible for the synthesis of the extracellular matrix, mainly composed of collagen in liver tissue, during fibrosis [3]. Upon liver injury, HSCs are known to become activated and transdifferentiate into proliferative, fibrogenic myofibroblasts and it is now well established that they are a central driver of fibrosis in experimental models and in human liver injury [4]. Recent studies based on single cell RNA sequencing (sc‐RNAseq) highlighted the co‐existence of different HSC subtypes during fibrogenesis with different activated ‘profiles’ such as inflammatory, proliferative or extracellular matrix producing, revealing the complexity of HSC activation and the phenotypic plasticity of HSC [5, 6, 7, 8]. These different subpopulations have been identified in different mouse models of fibrosis associated with MASH, CCl_4_ exposition or cholestasis (BDL model) as well as in human.

Moreover, the existence of two subpopulations of quiescent HSCs (qHSCs) expressing different genes depending on their location in the hepatic lobule, central vein‐associated HSCs (CaHSCs) and portal vein‐associated HSCs (PaHSCs), has recently been revealed in a murine model of fibrosis as well as in human liver [5, 6, 7, 8, 9].

In this context, we previously discovered in 4 murine models of liver fibrosis (dietary‐ or chemically‐induced) as well as in human liver biopsies with hepatic fibrosis due to different etiologies (alcoholism, MASH, or hepatitis C), the existence of a specific phenotype of hepatic stellate cells associated with collagen accumulation zones [10]. These cells exhibit a hypertrophied phenotype, with exacerbated accumulation of cytosolic droplets containing retinoids as we demonstrated that they emit vitamin A‐specific fluorescence. We called these cells hypertrophied hepatic stellate cells (hypHSC) and demonstrated that they express both markers of quiescent (desmin, cRBP1) and activated HSC (α‐SMA) [10]. Using a combination of fluorescent and non‐linear microscopies and the specific intrinsic retinoid droplet fluorescence, we were able to identify hypHSC in murine and human liver slices and determine their association with collagen deposition. In particular, we demonstrated a significant positive correlation between the stage of hepatic fibrosis and HSC hypertrophy in a cohort of obese patients with hepatic fibrosis.

We therefore hypothesize that hypHSCs constitute one of the hepatic stellate cell subpopulations that could play a direct or indirect role in liver fibrosis. Further characterization of these cells, such as analysis of their gene expression, appears necessary. So far, hypHSCs have been identified by microscopy analysis of murine liver slices and human liver biopsies with fibrosis. The next step is to produce a purified preparation of these cells for their molecular characterization.

The aim of this paper is to perform a transcriptomic analysis of hypHSCs isolated from fibrotic murine liver thanks to an experimental strategy we developed and described here. For this work, we have chosen the CDAHFD (choline deficient amino acid defined high fat diet) murine model we had previously used, since it is a murine model of MASH that recapitulates all the stages of the disease with steatosis, inflammation, progressive fibrosis associated with HSC hypertrophy with a very high reproducibility.

The hypHSC isolation strategy consists of two main steps: (1) hepatic dissociation and enrichment in HSCs by density gradient followed by (2) fluorescence‐activated cell sorting (FACS) thanks to hypHSCs or qHSCs intrinsic fluorescence. We also describe a characterization of hypHSCs at protein‐level marker expression, followed by transcriptomic analysis (bulk RNA‐seq), comparing their gene expression profile with that of qHSCs.

## 2 Materials and Methods

### 2.1 Animals and Experimental Design

C57BL/6 mice (Janvier Labs, France) were housed in cages (5 per cage) and were kept at 23+/−3°C with a 12:12 h light/dark cycle. Male 6‐month‐old (former breeders) or 6‐week‐old mice were fed a standard diet (SD, Specific Diet Services, England 841 201, vitamin A content 8000 IU/kg) for up to 6 months. After 5 days of acclimation, male 6‐week‐old mice were fed a choline‐deficient, 0.1% methionine, high‐fat diet (CDAHFD, Safe Diets, France U8958P Version 0247, vitamin A content 5280 IU/kg) for up to 20 weeks. Mouse weight, behavior and appearance were monitored weekly. All animal experiments have been approved by the Ethics Committee for Animal Experimentation and authorized by the French Ministry of Higher Education and Research (APAFiS #33532–2 022 030 115 518 551 v3 and #46428–2 023 092 921 282 299 v4).

### 2.2 HSC Isolation

#### 2.2.1 In Situ Enzymatic Dissociation of the Liver

Mice (SD or CDAHFD fed) were anesthetized with a mixture of ketamine/xylazine (180/20 mg/kg) by intraperitoneal injection. After checking for deep anesthesia by absence of paw withdrawal, a laparotomy was performed followed by lifting of costal plastron quickly after respiratory arrest. Perfusion of the liver through the inferior vena cava was then performed with a 21G needle (Venofix A, B. Braun) connected to pre‐heated (42°C) perfusion buffer (25 mM HEPES, 1.2 mM KH_2_PO_4_, 2.3 mM KCl, 86 mM NaCl, 0.5 mM EGTA, pH 7.6) at a rate of 4 mL/min. Then the portal vein was cut and perfusion continued with 45 mL of perfusion buffer at a flow rate of 4 mL/min. Liver perfusion was followed with 25 mL (SD mice) or 50 mL (CDAHFD mice) of pre‐heated (42°C) digestion buffer (25 mM HEPES, 2.3 mM KCl, 86 mM NaCl, 5 mM CaCl_2_, pH 7.6) containing Collagenase type I (Gibco 17 100–017) at 205 U/mL for (SD mice) or 375 U/mL (CDAHFD mice) and Dispase (Gibco 17 105–041) at 1 U/mL (SD mice) or 2 U/mL (CDAHFD mice) at a rate of 4 mL/min (SD mice) or 6 mL/min (CDAHFD mice). The liver was then removed, cut into small pieces, and incubated in 25 mL of digestion buffer, complemented with 250 μL DNAse I (2 mg/mL, Roche, 10 104 159 001), at 37°C for 20 min (SD mice) or 25 min (CDAHFD mice). The digested liver suspension was filtered through a 70 μm cell strainer (Falcon 352 350) and centrifuged at 600 g at 4°C for 15 min. Cells were then washed with 50 mL PBS complemented with 120 μL DNAse I, centrifuged at 600 g at 4°C for 15 min, and resuspended in 32 mL of Gey's Balanced Salt Solution (GBSS/B, 0.4 mM Na_2_HPO_4_, 2.7 mM NaHCO_3_, 0.2 mM KH_2_PO_4_, 137 mM NaCl, 5 mM KCl, 1 mM MgCl_2_, 0.3 mM MgSO_4_, 5.5 mM Glucose, 2 mM CaCl_2_, pH 7.4) complemented with 120 μL DNAse I. Antibiotics were added to buffers (except for perfusion buffer) of all steps of cell preparation from CDAHFD‐mouse liver (dissociation, gradient step, sorting, and immunolabeling): penicillin (200 U/mL), streptomycin (200 μg/mL) (Gibco, 15 140–122), and gentamicin (70 μg/mL) (Gibco, 15 710–049).

#### 2.2.2 HSC Isolation Using a Density Gradient

Cells suspended in GBSS/B were mixed with 16 mL Nycodenz solution (Serumwerk, 18 003; 0, 3 g/L in GBSS/A) and then divided into 4 × 15 mL‐tubes, each containing 12 mL of the cell/Nycodenz mixture. GBSS/A buffer has the same composition as GBSS/B buffer without addition of NaCl. A volume of 1.5 mL of GBSS/B buffer was gently added on top of the cell/Nycodenz mixture, using a syringe connected to 26G needle. The tubes were centrifuged at 1380 g at 4°C for 17 min without brake. HSCs were collected at the interface formed between GBSS/B and GBSS/A washed in PBS, centrifuged at 600 g for 15 min at 4°C and resuspended in 500 μL PBS supplemented with 1% FBS (Fetal Bovine Serum). Cells were counted using a Malassez cell.

#### 2.2.3 HSC Sorting Using FACS

The cell concentration was adjusted to 10^7^ cells/mL in PBS‐FBS 1% and cell sorting was performed with the MoFlo Astrios (Beckman Coulter, France) at 25 PSI with a 100 μm nozzle at approximately 4000 events per sec. The FSC and SSC were read logarithmically with the 488 nm laser and the threshold has been set on the FSC. For hypHSC sorting, green fluorescence was read with the 488 laser (513/26 band pass classically used for FITC or GFP) and for qHSC sorting, blue fluorescence was read with the 405 nm laser (448/59 band pass classically used for BV 421). The sorting gate of hypHSC was defined on SSC‐561 on linear scale vs. green fluorescence 488 laser (513/26 band pass) plot for better definition.

In our experience, the number of sorted events given by sorter was always greater than the number of cells, meaning that this number corresponded rather to the number of elements detected by the device than the actual number of cells. Thus, a first sorting of 100 000 events was carried out, to count the number of cells obtained using a Mallassez cell. A % of events corresponding to cells was then obtained and made it possible to determine the number of events to be collected to actually have the number of cells required. For example, if cell sorter indicated that 100 000 events were sorted, and we counted 40 000 cells, we assumed that 40% of the events detected by the sorter corresponded to actual cells. NB: We observed that a % greater than 60% of events corresponding to cells gave a good quality cell preparation.

For qPCR analysis, between 100 000 and 300 000 sorted HSCs were suspended in RNA later (Invitrogen, AM7020), flash‐frozen in dry ice and stored at −80°C. For immunofluorescent labeling experiments, sorted HSCs were suspended in PBS‐FBS 10% or DMEM containing 10% FBS.

For RNA‐seq analysis, 20 000 sorted HSCs were harvested directly from the cell sorter in lysis buffer (Buffer RL #51800, Norgen Biotek Corp.), flash‐frozen in dry ice, and stored at −80°C.

### 2.3 Microscopic Analysis of Cells After Gradient or After Sorting

HSC quantification was performed by counting fluorescent cells on images acquired with an emCCD camera Retiga 2000 (Photometrics) mounted on an Axiovert (Zeiss, Germany) microscope and analyzed using ImageJ software. Filter set configuration was as follows: blue fluorescence for qHSCs from SD‐mouse liver: excitation: 365/12 nm and emission long pass starting at 397 nm; green fluorescence for hypHSCs from CDAHFD‐mouse liver excitation: 470/40 nm and emission: 540/50 nm. Objectives used were 32X/NA 0,4 and 20X/NA 0,3 (Zeiss, Germany).

The number of total cells was counted under phase contrast illumination and the number of HSCs was determined by counting cells containing droplets emitting blue‐only (qHSC) or blue and green (hypHSC) fluorescence on at least 5 microscope's fields corresponding to 100–200 cells for each condition. The purity of hypHSCs was determined by calculating the ratio of cells containing green fluorescence droplets to total cells. The purity of qHSCs was determined by calculating the ratio of cells containing blue fluorescence droplets to total cells. For sorted hypHSCs, the number of intact hypHSCs was determined by counting green fluorescent cells with fluorescence contained in intact cytoplasmic droplets, and the number of opaque hypHSCs was determined by counting the number of cells showing fluorescence throughout the cell cytoplasm. The percentage of intact hypHSCs was determined by calculating the ratio of the number of intact hypHSCs counted to the number of total cells, and the percentage of opaque hypHSCs was determined by calculating the ratio of the number of opaque hypHSCs counted to the number of total cells.

#### 2.3.1 Immunostaining of Sorted Cells

Sorted HSCs were resuspended in DMEM medium (10% FBS) and seeded in microwells mounted on a polymer coverslip (Ibidi) and covered with collagen gel (Collagen type I, 1.5 mg/mL final in NaOH 6.7 mM) at a density ranging from 20 000 to 50 000 cells/cm^2^. Cells were incubated at 37°C and 5% CO_2_ for 1–2 days, to enable their adhesion to the collagen gel and avoid cell loss during the next steps. The cells were then fixed with 4% para‐formaldehyde for 30 min at room temperature (RT), rinsed with 1% PBS‐BSA, permeabilized (only in the case of intracellular protein labeling) with 0.1% PBS‐Triton X100 for 10 min at RT. After rinsing with 1% PBS‐BSA, cells were incubated for at least 1 h in 1% PBS‐BSA and then incubated with primary antibodies diluted in 1% PBS‐BSA and 0.05% Triton X100 overnight at 4°C. After rinsing with 1% PBS‐BSA, cells were incubated with secondary antibodies and DAPI (Sigma‐Aldrich, D1388, 1/1000) diluted in 1% PBS‐BSA and 0.05% Triton X100 for 1 h at RT. Primary antibodies were used at the following dilutions: cRBP1 (abcam ab154881; 1/50), anti‐desmin conjugated to AlexaFluor647 (Santa Cruz Biotechnology sc‐23 879; 1/10); LRAT (ab166784; 1/20), αSMA (A5228; 1/10), anti‐CD36 (Miltenyi 130–122‐091, 1/50) and anti‐CD68 (Miltenyi 130–112‐857, 1/50) and secondary antibody Goat anti‐rabbit IgG conjugated to Alexa Fluor 647 (Invitrogen, A‐21244; 1/200). Cells were then rinsed with 1% PBS‐BSA and mounted in mounting medium (Fluoromount‐G; Invitrogen) up to 24 h at 4°C. Microscopy acquisitions were performed with laser scanning fluorescent microscope SP8 (Leica Microsystems, Germany) using a 40×/NA 1,3 oil immersion objective. Images were acquired using the software Leica Acquisition System X (Leica Microsystems, Germany) and treated using open source FiJi software.

### 2.4 Analysis of Cells After Gradient Using Flow Cytometry

Cells recovered after the gradient were suspended in PBS‐FBS 10% at a density of 30 000 to 50 000 cells/100 μL and fixed with 2% PBS‐PFA for 15 min at RT. After washing by adding 200 μL PBS‐FBS 10% and centrifugation at 600 g at 4°C for 15 min, they were resuspended at the same density in 10% PBS‐FBS and primary and secondary antibodies were added. If the antibody targets an intracellular protein, cells were previously permeabilized by adding 0.05% Triton X100. Tubes were gently shaken by rotation for 40 min at RT. Cells were then washed by adding 200 μL of 10% PBS‐FBS, centrifuged at 600 g at 4°C for 15 min and were resuspended in 10% PBS‐FBS at an average of 30 000 cells/100 μL for flow cytometry analysis. The following antibodies were used: at 1/50 dilution, anti‐ASGPR 1/2 FL‐291; (sc‐28 977), anti‐RBP1 (ab154881), anti‐Desmin (ab15200), anti‐CD36 (Miltenyi, 130–122‐091) and anti‐CD68 (Miltenyi, 130–112‐857), and at 1/200 dilution, anti F4/80 (CI:A3‐1) (sc‐59 171) and anti‐CD31 (MEC 13.3) (BD Pharmingen, 550 274). The following secondary antibodies were used: Goat anti‐rabbit IgG, Alexa Fluor 488, (Invitrogen, A‐11008) at 1/200 and Goat anti‐rat IgG, Alexa Fluor 488, (Invitrogen, A‐11006) at 1/400. Labeling controls were performed using the same protocol, without addition of primary antibody and only addition of secondary antibody at the corresponding step. Cell suspensions were analyzed on a flow cytometer Guava Easycyte cytometer (Millipore) equipped with two lasers (excitation 488 nm and 640 nm). The percentage of labeled cells was calculated as the percentage of positive cells minus the percentage of nonspecific labeling found in the secondary antibody control (see Figures S3 and S4 for illustration of flow cytometry raw data). Unstained cells recovered after gradient were analyzed on a LSRII BD FACS Fortessa flow cytometer (BD Biosciences, Cybio facility, Cochin Institute) equipped with 4 lasers (excitation 355 nm, 405 nm, 488 nm and 640 nm). Data were processed using FlowJo software (version 10, Tree Star Inc.).

### 2.5 Analysis of Hepatic and Fecal Bacterial Load in SD and CDAHFD Mice

Mouse fecal pellets were collected at 0, 3, 6, 9 and 12 weeks of diet from CDAHFD fed mice (*n* = 4 to 8) and SD fed mice (*n* = 5), flash‐frozen and stored at −80°C. Liver frozen samples were lysed for 2 h in solution containing 25 μL of proteinase K (Qiagen) and 180 μL of ATL lysis buffer (Qiagen). Genomic DNA (gDNA) from fecal samples and liver tissue lysates was extracted according to the recommendations of the International Human Microbiome Standards (IHMS: http://www.human‐microbiome.org/). Briefly, aliquot of each sample was suspended in 250 μL of guanidine thiocyanate, 40 μL of 10% N‐lauroyl sarcosine, and 500 μL of 5% N‐lauroyl sarcosine. Genomic DNA was extracted by mechanical disruption of the microbial cells with glass beads (0.1 mm, Sigma‐Aldrich) using a TissueLyser III (QIAGEN), and nucleic acids were recovered from clear lysates by alcohol precipitation as previously described [11]. The concentration and purity of each gDNA sample obtained were assessed using a nanodrop (Nanodrop One, Thermo Fisher Scientific) and the quality of the DNA was assessed by means of a genomic gel (1% agarose, 100 V, 1 h) as recommended by the IHMS. Any sample showing poor purity or excessive degradation was excluded from the study.

Extracted genomic DNA was used to amplify the V4 region of the 16S rRNA gene by standard and quantitative real‐time PCR, using universal primers for counting the microbial load. Universal primers located in highly conserved sequences targeting the V4 hypervariable region of the 16S rRNA genes (290 bp) were used: V4F_517_17 (5‐GCC AGC CGC GGT AA‐3) and V4R_805_19 (5‐GAC TAC CAG GGT ATC TAA T‐3).

Standard PCR 0.15 units of Taq polymerase (AmpliTaq Gold, Life Technologies) and 20 pmol/μL of the forward and reverse primers (IDT Technologies) was run in a Mastercycler gradient (Eppendorf) at 94°C for 3 min, followed by 35 cycles of 94°C for 45 s, 56°C for 60 s, 72°C for 90 s and a final cycle of 72°C for 10 min.

In order to assess the microbial load, the extracted DNA was used to amplify the V4 region of the 16S rRNA gene by quantitative real‐time PCR (qPCR) using universal primers for counting microbial load as previously described [12]. The PCR was performed in a volume of 25 μL using Power SYBR green PCR master mix (Fisher Scientific) containing 100 nM (each) primer. The reaction conditions were 50°C for 2 min, 95°C for 10 min, and 40 cycles of 95°C for 15 s and 60°C for 1 min. All samples were analyzed in triplicate and mean values were calculated. Reading and analysis of results was evaluated using Bio‐Rad CFX Manager 3.1 software (Bio‐Rad).

### 2.6 Transcriptomic Analysis

#### 2.6.1 Bulk RNAseq

RNA was extracted from sorted qHSC or hypHSC using the Single Cell RNA purification (kit #51800, Norgen Biotek Corporation, Thorold, ON, Canada) according to the manufacturer's protocol and eluted in 23 μL. After extraction, RNA concentrations were obtained using the fluorometric Qubit RNA assay (Life Technologies, Grand Island, New York, USA). The quality of the RNA (RNA integrity number) was determined on the Agilent 2100 Bioanalyzer (Agilent Technologies, Palo Alto, CA, USA) as per the manufacturer's instructions using pico chip kit.

To construct the libraries, 2.5 ng of high‐quality total RNA sample (RIN > 6) was processed using NEBNext low input RNA library prep kit (NEB) according to manufacturer instructions. Briefly, total RNA molecules were reverse‐transcribed using oligo dT primers and template switching oligos. cDNA was then amplified with 3 and 5 adapters and QC controlled. After fragmentation, amplified cDNA was then end repaired and ligated with NEBNEXT adapters. PCR enrichment with single barcode was then realized to obtain the final library. Libraries were then quantified with Qubit HS DNA assay (Life Technologies, Grand Island, New York, USA) and library profiles assessed using the DNA High Sensitivity LabChip kit on an Agilent 2100 Bioanalyzer (Agilent Technologies, Palo Alto, CA, USA). Libraries were sequenced on an Illumina Nextseq 2000 instrument using 59 base‐lengths read V2 chemistry in a paired‐end mode.

RNA‐Seq data analysis was performed by GenoSplice technology (www.genosplice.com). Analysis of sequencing data quality, reads repartition (e.g., for potential ribosomal contamination), inner distance size estimation, genebody coverage, strand‐specificity of library were performed using FastQC v0.11.2, Picard‐Tools v1.119, Samtools v1.0, and RSeQC v2.3.9. Reads were mapped using STAR v2.7.5a [13] on the Mouse mm39 genome assembly and read count was performed using featureCount from SubRead v1.5.0 and the Mouse FAST DB v2022_1 annotations. Gene expression was estimated as described previously [14]. Only genes expressed in at least one of the two compared conditions were analyzed further. Genes were considered as expressed if their FPKM value was greater than FPKM of 98% of the intergenic regions (background). Analysis at the gene level was performed using DESeq2 [15]. Genes were considered differentially expressed for log2FC ≥ 1 and adjusted *p ≤* 0.05. Overrepresented analyses were performed using WebGestalt v0.4.4 [16] merging results from up‐regulated and down‐regulated genes only, as well as all regulated genes. Pathways and ontologies were considered significant with *p ≤* 0.05.

#### 2.6.2 qPCR

Cells sorted and recovered in RNA later were centrifuged at 5000 g at 4°C for 10 min. RNA was then extracted using the RNeasy kit (Qiagen, 741 004) according to provider's recommendations and eluted in 30 μL of RNAse‐free water. Extracted RNA was first treated with DNAse I (Takara, 2270A) to remove genomic DNA, and complete removal was controlled by PCR. RNA concentration was then determined using the Quant‐iT RiboGreen RNA kit and reagent (Invitrogen, R11490). 10 pg to 5 μg of RNA were mixed with 1 μL of 50 μM random primers, 1 μL of 10 mM dNTP and RNAse‐free water to give a final volume of 13 μL and incubated at 65°C for 5 min to allow primer hybridization, then on ice for 1 min. 4 μL of SSIV Buffer, 1 μL of 100 mM DDT, 1 μL of RNAse OUT (Invitrogen, 10 777 019), and 1 μL of Reverse Transcriptase SuperScript IV enzyme (SSIV, Invitrogen, 18 090 010) were added to the mixture, which was then incubated at 23°C for 10 min, then at 55°C for 10 min to allow cDNA synthesis, and finally at 80°C for 10 min to inactivate the enzyme. cDNAs were stored at −80°C.

For qPCR analysis, 1 μL of diluted cDNA (1:10 in RNAse‐free water) was deposited in 384‐well microplate to which was added 19 μL of a mixture containing: 10 μL of Power SYBER Green Master Mix (Thermo Fisher Scientific, 10 219 284), 0.1 μL of the sense strand primer, 0.1 μL of the anti‐sense strand primer (See Table S3), and 8.8 μL of RNAse‐free water. qPCR was performed on the BIO‐Rad CFX 384, according to the following steps: enzyme activation by heating the plate at 95°C for 10 min, followed by 40 cycles of denaturation at 95°C for 15 s and hybridization and elongation at 60°C for 1 min. Results were analyzed using Bio‐Rad CFX Maestro software. The stability of housekeeping genes was assessed on samples of sorted hypHSCs and qHSCs, using the GeNorm algorithm of the “Reference Gene Selection” tool in Biorad's CFX software. This analysis validated 18S, HPRT1, Actin, and PGK1 as the 4 reference genes for studying the expression of genes of interest in different HSC populations.

## 3 Results

### 3.1 Enriched hypHSC Preparation From CDAHFD‐Mouse Liver

#### 3.1.1 Protocol Design

**FIGURE 1:**
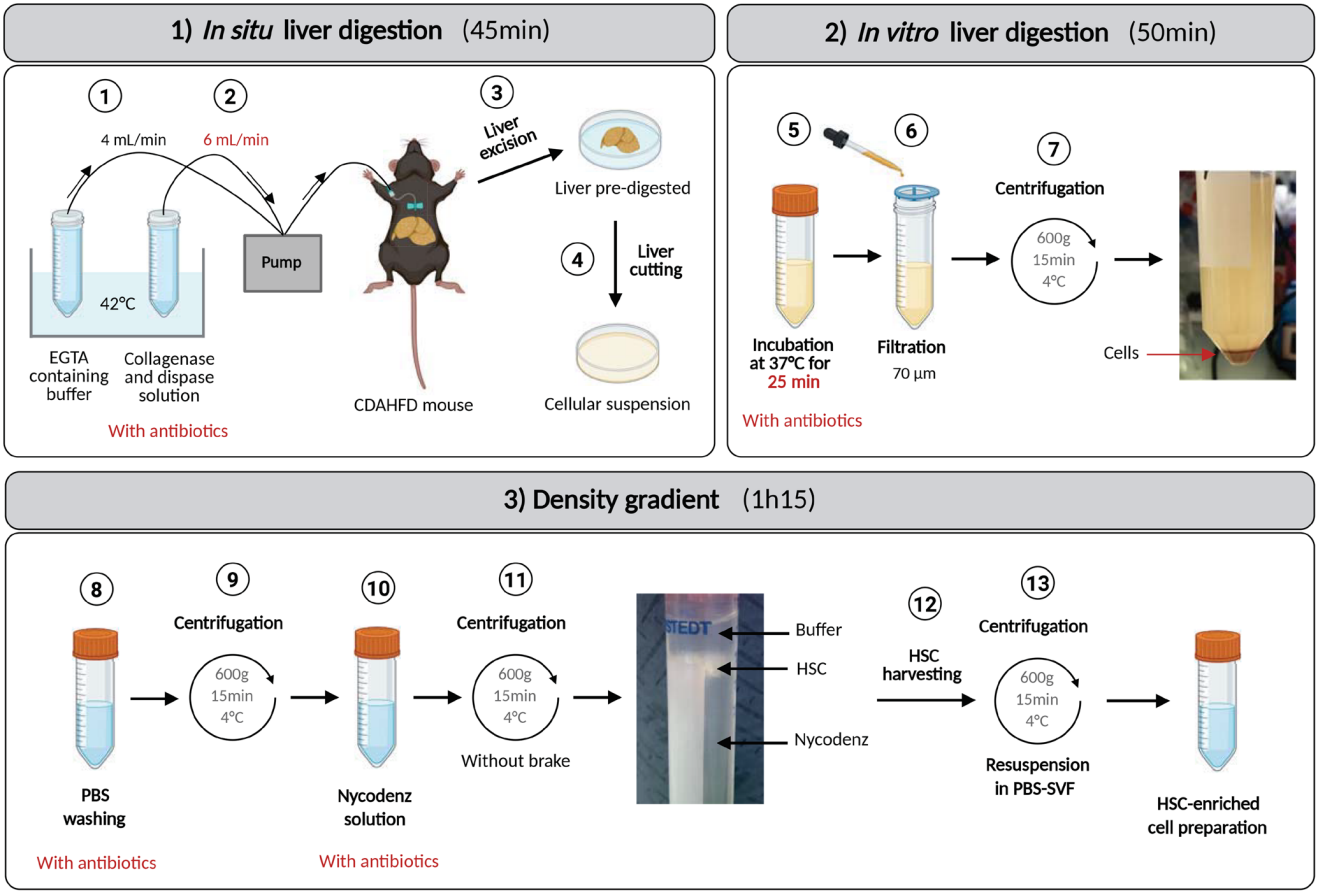
Schematic representation of the steps involved in the purification of hypertrophied HSCs from CDAHFD mice. Changes in conditions compared with the purification of quiescent HSCs from SD mice are highlighted in red. (1) Perfusion of the inferior vena cava by insertion of a 21G needle connected to a pump with perfusion buffer (containing EGTA) at 4 mL/min. (2) Perfusion of the liver with a buffer containing enzymes (collagenase and dispase) buffer and antibiotics (penicillin, streptomycin and gentamicin) and increased perfusion rate to 6 mL/min. (3) Excision of the digested liver and transfer to a Petri dish containing buffer with enzymes and antibiotics. For experiments requiring sterile conditions, manipulate within a biosafety cabinet from this stage onwards. (4) Cutting of the liver to release the cells. (5) Incubation of the resulting cell suspension at 37°C for 25 min to continue in vitro digestion. (6) Filtration of the cell suspension through a 70 μm‐filter to remove undigested liver pieces. (7) Centrifugation to pellet the cells and to remove enzyme buffer. (8) Rinsing with PBS containing antibiotics. (9) Centrifugation to pellet the cells and remove PBS. (10) Mixing of the cell suspension with a Nycodenz solution. (11) Centrifugation to form the density gradient to separate HSCs, thanks to their low density, from other liver cells. (12) Harvesting HSCs floating on the gradient. (13) Centrifugation to remove buffer and resuspend the cells in PBS‐FBS 1%. Created in BioRender. Hoffmann, C. (2025) https://BioRender.com/im0zs9r.

Our starting point for hypHSC isolation relied on the well‐described isolation protocol described by Mederacke et al. (Figure 1). This method consists of in situ dissociation of the liver by enzyme perfusion, followed by excision of the liver to recover the cells as a suspension and by density gradient for separation of high‐lipid‐content cells [17, 18, 19] (Figure 1).

In CDAHFD‐murine model, hypHSCs start to be detected from 6 weeks of CDAHF diet (see Figure S1). At this point, the liver exhibits stage F1 fibrosis and stage 3 steatosis, requiring adaptation of the protocol to this collagen‐ and lipid‐laden pathological microenvironment. Isolation of hypHSCs was performed from the liver of CDAHFD‐fed mouse for at least 9 weeks of diet, as the hypHSC content is stable from this point onwards. Quiescent HSCs, that were considered as control cells, were isolated from the liver of mouse fed with a standard diet (SD), between 6 and 10 months old to recover sufficient HSCs, as qHSC level increases with aging [20, 21].

Firstly, the conditions of CDAHFD‐mouse liver digestion were adapted from the qHSC isolation protocol, by increasing (i) the enzyme concentrations (×1.5 for collagenase, ×2 for dispase), (ii) the perfusion flow rate (from 4 to 6 mL/min), (iii) the digestion buffer volume (×1.5) and (iv) the post‐perfusion incubation time (+5 min). Secondly, the centrifugation steps were adjusted by resuspending the cells in PBS for washing before resuspending them in GBSS/B buffer for the gradient step, as we observed a loss of cells when washing in GBSS/B directly. Remarkably, after the first centrifugation step, we observed for CDAHFD‐mouse liver cell suspension the formation of a fat layer on the surface of the supernatant that is not observed for SD‐mouse liver cell suspension. This is due to the steatotic state of CDAHFD‐mouse liver. After this first centrifugation step, the layer of fat was carefully removed before continuing the experiment.

Another major modification to the protocol was the addition of antibiotics (Penicillin/Streptomycin/Gentamycin) in each buffer since systematic bacterial contamination was observed in cell preparations from CDAHFD‐mice liver, whereas this was never the case from SD‐mice liver, despite the same precautions for sterility being taken. We hypothesized that contamination of CDAHFD preparation was due to an endogenous contamination of CDAHFD liver. Some authors described that under fat diet, an intestinal bacterial translocation may occur [22, 23]. To ensure this hypothesis, we assessed the bacterial load in the liver and feces of CDAHFD‐ and SD‐mouse using classical and real‐time PCR amplification of the 16S rRNA gene. We observed a decrease in bacterial load in CDAHFD‐mice feces and an increase in bacterial load in CDAHFD‐mouse liver, as compared with samples from control mice. These observations could be linked to a relocation of bacteria from the gut to the liver, suggesting a phenomenon of bacterial translocation in our model (See Figure S2).

#### 3.1.2 Cell Characterization After the Gradient Step

**FIGURE 2:**
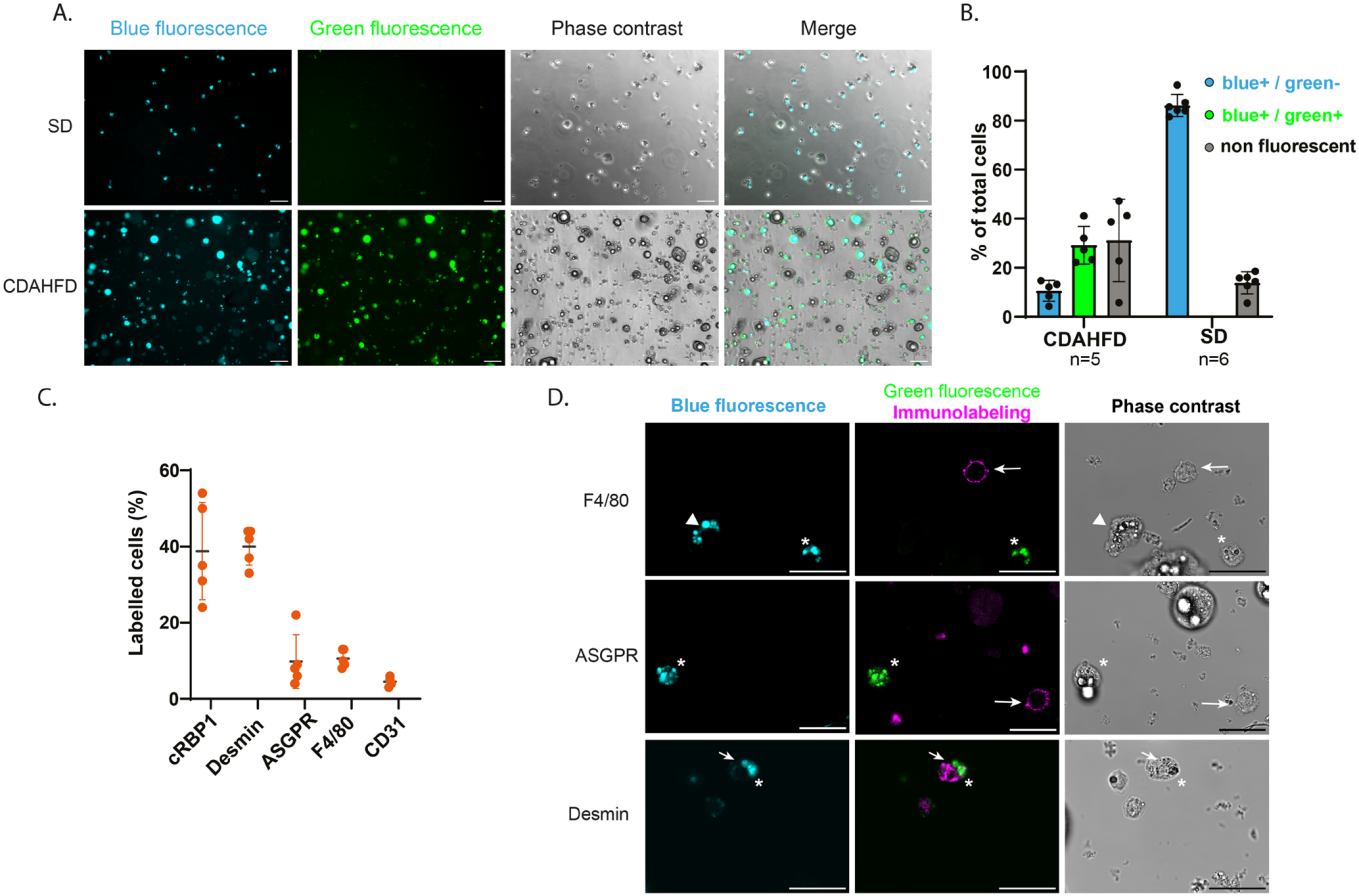
Characterization of cells obtained after density gradient. (A) Representative microscopy images of cell preparation from CDAHFD‐ and SD‐ liver. Acquisitions under UV–Vis excitation for blue fluorescence (λ_exc_ = 365 nm λ_em_ > 390 nm), under excitation at 488 nm for green fluorescence (λ_exc_ = 488 nm, λ_em_ = 500–550 nm), phase contrast illumination, and merge of images. Scale bar: 50 μm. (B) Percentage of fluorescent cells from CDAHFD‐ and SD‐mouse liver counted from microscopic images. (CDAHFD, *n* = 5 and SD, *n* = 6 independent experiments) (C) Repartition of hepatic cell population in CDAHFD cell preparation using cytometry analysis. Percentages of labeled cells were obtained after immunolabeling of cells with typical markers of HSCs (cRBP1 and desmin, *n* = 5), hepatocytes (ASGPR, *n* = 5), macrophages (F4/80, *n* = 5) and endothelial cells (CD31, *n* = 4). (D) Representative microscopy images of cell preparation after density gradient from CDAHFD‐liver after immunostaining with ASGPR, F4/80 or desmin antibodies (magenta, λ_exc_ = 638 nm, λ_em_ = 650–690 nm) and acquisitions for blue fluorescence (λ_exc_ = 405 nm, λ_em_ = 425–465 nm, cyan) and green fluorescence (λ_exc_ = 488 nm, λ_em_ = 505–535 nm, green). Arrowhead: qHSC, star: hypHSC, arrows: cells with positive corresponding immunolabeling. Scale bar = 25 μm.

After density gradient, 6.4 ± 4.1 x 10^6^ cells (*n* = 53) were recovered from CDAHFD‐mice liver (9–15 weeks of diet) vs. 2.9 ± 2.0 x 10^6^ cells (*n* = 16) from SD‐mice liver (5–10 months old). Thus, on average, 2.2 times more cells are recovered from the liver of CDAHFD‐mice than from SD‐mice (Table 1). This difference is statistically significant (*p* = 2.10^−4^, Mann–Whitney test) and probably stems from the fact that the cell populations present in CDAHFD‐mice liver differ from those in SD‐mice liver in terms of distribution and characteristics (steatotic hepatocytes, several hepatic stellate cell phenotypes), as a greater cellular diversity is observed in post‐gradient CDAHFD‐mice liver preparations (Figure 2A).

**TABLE 1:** Number of cells obtained at the different stages of HSC purification.

Diet	Average number of cells		% of cells sorted versus cells after gradient	% of dead cells	% of hypHSC or qHSC purity
	After gradient	After sorting			
CDAHFD (9–15 weeks)	6.4 ± 4.1 × 10^6^ (*n* = 53)	2.4 ± 1.1 × 10^5^ (*n* = 30)	3.5% ± 1.1% (*n* = 30)	22.0% ± 14.0% (*n* = 25)	94.8% ± 4.5% (*n* = 19)
SD (5–10 months)	2.9 ± 2.0 × 10^6^ (*n* = 16)	4.4 ± 3.0 × 10^5^ (*n* = 13)	21.6% ± 9.1% (*n* = 13)	12.2% ± 8.7% (*n* = 10)	94.0% ± 3.2% (*n* = 8)

*Note:* Cells were counted manually using Mallassez cells. Dead cells were quantified using Trypan Blue coloration. For CDAHFD‐mouse liver, *n* = 53 liver dissociations followed by density gradient were performed. Among them, 30 have been followed by FACS. Percentage of dead cells and hypHSC purity were quantified for 25 and 19 independent hypHSC sorted preparations respectively. For SD‐mouse liver, *n* = 16 liver dissociations followed by density gradient were performed. Among them, 13 have been followed by FACS. Percentage of dead cells and qHSC purity was quantified for 10 and 8 independent qHSC sorted preparations respectively.

One of the specific characteristics of HSCs is that they contain cytoplasmic droplets rich in retinoids. These retinoids emit a specific endogenous fluorescence in the near UV‐blue range, with excitation between 365 and 405 nm and fluorescence emission from 380 to 500 nm [24, 25]. Previously, we highlighted on CDAHFD‐mouse liver sections that hypHSCs exhibit specific and broad fluorescence properties, with a fluorescence excitation in the UV–visible spectrum and emission throughout the visible spectrum (400–600 nm). Notably, hypHSCs retain the fluorescence properties in the UV‐violet range like qHSC (λ_exc_ = 365 nm and λ_em, max_ = 390 nm) and, after excitation at 488 nm, emit an additional intense signal in the green range (500–550 nm), while this is not the case for qHSCs, which are not excitable beyond 405 nm [10].

In this study, we used these specific fluorescence properties to identify hypHSCs and qHSCs in cell preparations. We qualified the natural fluorescence of qHSC (λ_exc_ = 365 nm, λ_em, max_ = 390 nm) as “blue” fluorescence and the specific hypHSC fluorescence in the green range (λ_exc_ = 488 nm, λ_em, max_ = 525 nm) as “green” fluorescence. Therefore, qHSCs are blue+/green‐ and hypHSCs are blue+/green+ (Figure 2A).

As shown in Figure 2B, after the gradient, the cell preparation from CDAHFD‐mouse liver contained approximately 1/3 hypHSCs (blue+/green+) as well as other cells containing retinoids (blue+/green‐) that may be remaining qHSCs and also a majority of non‐fluorescent cells. The preparation from a SD‐mouse liver did not contain hypHSCs, only a large majority of qHSCs (blue+/green‐) and a few non‐fluorescent cells.

The composition of cell population from CDAHFD‐mouse liver was further analyzed by flow cytometry after immunolabeling with antibodies specific to liver cells (CD31, endothelial cells; ASGPR, hepatocytes; F4/80, Kupffer cells; desmin and cRBP1, HSCs). As shown in Figure 2C, we found 40.0% ± 4.8% of cells that were desmin+, and 38.8% ± 12.7% that were cRBP1+, while the other antibodies did not label more than 10% of cells, confirming the enrichment of the preparation in HSCs (see Figures S3 and S4 for illustration of flow cytometry raw data). Fluorescence microscopy imaging allowed us to better characterize the cell population obtained after density gradient (Figure 2D). qHSCs were identifiable thanks to blue fluorescence contained in cytoplasmic droplets and hypHSCs exhibited, as previously described, blue and green fluorescence in cytoplasmic droplets. Immunolabeling of ASGPR and F4/80 was observed around cell membrane of non‐fluorescent cells and never colocalized with cells containing fluorescent droplets. Unlike ASGPR or F4/80, desmin immunolabeling was colocalized with cells containing blue and blue/green cytoplasmic droplets corresponding to qHSCs and hypHSCs, respectively (stars and arrowheads on Figure 2D).

Thus, as hypHSCs represented only 30% of the cell population obtained after gradient from CDAHFD‐mouse liver, which was not sufficient for further characterization of these cells, an additional purification step was essential. To obtain a highly pure hypHSC preparation, we used fluorescence‐assisted cell sorting (FACS), taking advantage of the specific fluorescence properties of these cells, thus allowing sorting to be carried out without requiring any exogenous labeling.

### 3.2 Sorting of hypHSC

#### 3.2.1 Sorting Strategy

**FIGURE 3:**
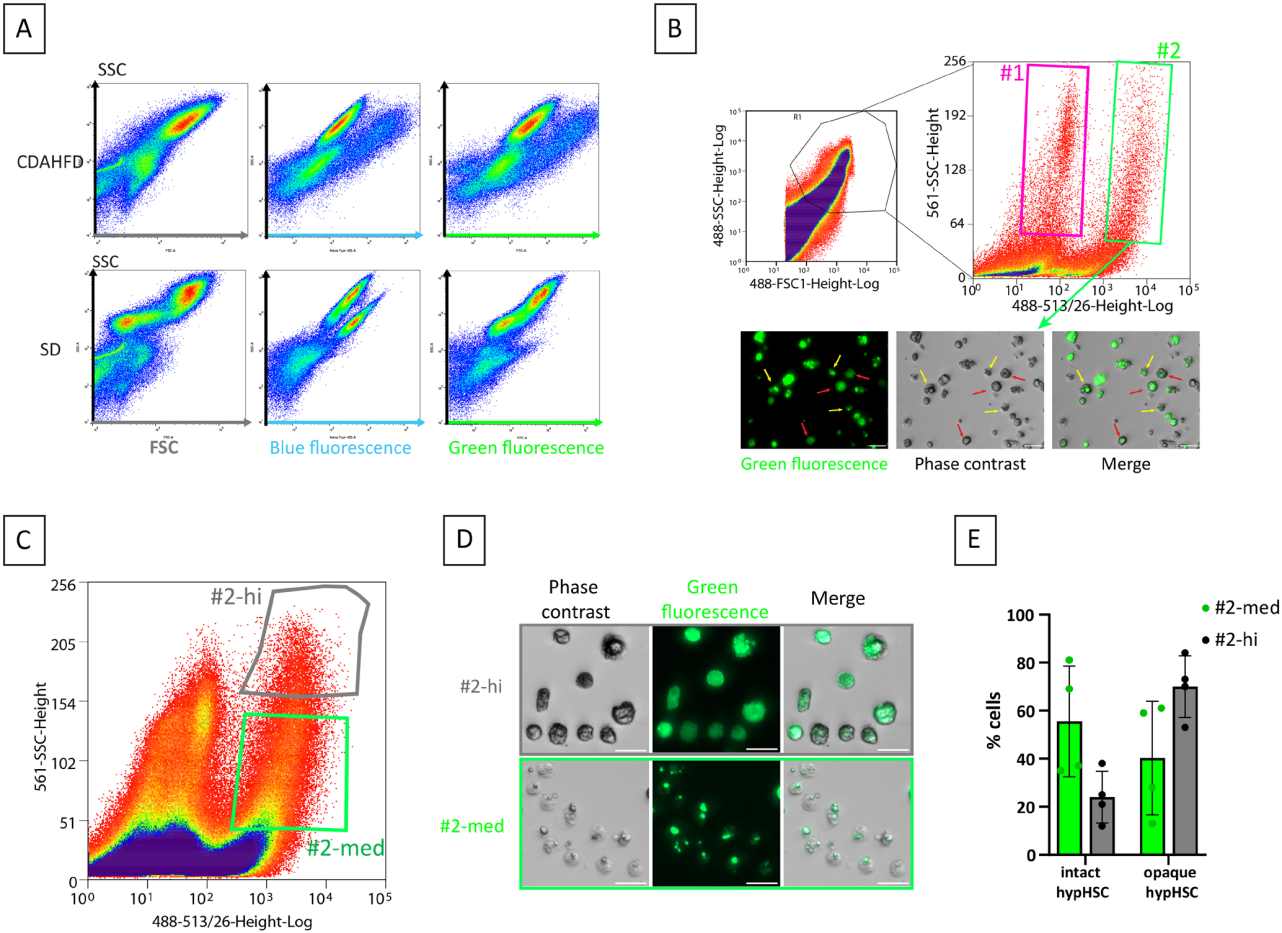
Strategy for hypHSC fluorescence‐activated cell sorting and characterization by fluorescence microscopy. (A) Cytometric analysis of cell preparations obtained from CDAHFD‐ and SD‐mice liver after density gradient. Dot plot of the CDAHFD and SD cell preparation analyzed for FSC/SSC (left panels), SSC and blue fluorescence (λ_exc_ = 405 nm and λ_em,max_ = 450 nm; middle panels), SSC and green fluorescence (λ_exc_ = 488 nm and λ_em,max_ = 530 nm; right panels). (B) Dot plot of the CDAHFD cell preparation for FSC/SSC (left panel) and for SSC (linear)–green fluorescence (λ_exc_ = 488 nm and λ_em,max_ = 530 nm; right panel). Gate #1 corresponds to low fluorescent cells and gate #2 corresponds to highly fluorescent cells. Below panels: Phase contrast and green fluorescence (λexc = 488 nm, λem = 500–550 nm) microscopy images corresponding to gate #2. Red arrows point opaque hypHSCs and yellow arrows point intact hypHSCs. Scale bars = 25 μm. (C) Dot plot of the CDAHFD cell preparation for SSC (linear)–green fluorescence (λ_exc_ = 488 nm and λ_em,max_ = 530 nm). Gate #2‐hi and #2‐med correspond to fluorescent cells with high granularity and medium granularity, respectively. (D) Phase contrast and green fluorescence (λ_exc_ = 488 nm, λ_em_ = 500–550 nm) microscopy images corresponding to gates #2‐hi and #2‐med highlighting opaque and intact hypHSC phenotypes. Bars = 25 μm. (E) Quantification of opaque and intact hypHSCs by microscopy as a function of sorting gate. Means ± standard deviation are shown (*n* = 4 independent experiments).

The cell preparation obtained after density gradient contains a mixture of cells of varying size and granularity, and probably cell debris. Cytometric analysis for both parameters (FSC and SSC) was not sufficient to identify with certainty the position on the cloud corresponding to the viable cells. Cell labeling with SYTO16 allowed us to determine the position of intact cells cloud on the FSC‐SSC dot‐plot (See Figure S3). Thus, after an initial selection of intact cells on the FSC‐SSC dot‐plot, the cells were analyzed according to SSC and green fluorescence (λ_exc_ = 488 nm, λ_em_ = 513/26 nm) or blue fluorescence (λ_exc_ = 405 nm and λ_em_ = 425–475 nm) (Figure 3A). We then observed, for both CDAHFD and SD cell preparations, the presence of a blue‐fluorescing cell population corresponding to qHSCs. And as expected, a population of cells emitting green fluorescence was found in the CDAHFD cell preparation but not in the SD cell preparation, so we assumed this part of the cloud to be hypHSCs (Figure 3A).

Starting from these analyses, the hypHSC sorting strategy was based on the unique green fluorescence of hypHSCs preceded by a succession of sorting gates. Sorting was carried out on an Astrios equipped with a 100 μm nozzle, allowing the sorting of cells from liver cell preparation (containing large cells) with a relatively low pressure flow regarding the fragility of hypHSCs. First, cell selection was performed on the FSC–SSC dot‐plot to eliminate cellular debris; then, a selection of single cells was made to eliminate doublets; finally, the cell cloud was selected on the green fluorescence signal to recover hypHSCs (Figure 3B). To select hypertrophied HSCs, the SSC signal on the 488 laser was switched to SSC on the 561 laser to obtain an SSC signal free from the fluorescence contribution of retinoid droplets from hypHSCs at 488 nm. Second, the SSC‐561 had to be expressed on a linear scale to obtain a more spread‐out distribution of points that allowed for a better definition of the population (Figure S5).

Different gates for recovering the cells were determined on the SSC‐lin/green‐fluorescence plot and the morphology of the cells was analyzed by fluorescence microscopy to determine the best gate for hypHSC sorting (Figure 3B). As expected, cells from gate #1 characterized by low fluorescence signal (between 10^1^ and 10^2^) corresponded to non‐fluorescent cells under fluorescence microscopy while the cells from gates #2, characterized by a high green fluorescent signal (between 2.10^2^ and 10^4^) exhibited fluorescent droplets after excitation at 488 nm detectable by fluorescence microscopy corresponding to hypHSCs (Figure 3B).

Microscopic observations showed that the hypHSC population recovered from gate #2 was of heterogeneous phenotype. Part of cells exhibited fluorescence restricted in well‐defined fluorescent droplets, while another part exhibited a very bright fluorescence throughout the cytoplasm (Figure 3B). We called cells still exhibiting fluorescent droplets “intact” hypHSCs and cells no longer exhibiting droplets but retaining intense residual fluorescence “opaque” hypHSCs, because they appeared opaque under phase‐contrast illumination (Figure 3B).

We chose to harvest hypHSCs with intact retinoid droplets, as this is the form in which we have previously identified them in CDAHFD‐mice liver sections and human biopsies [10]. Furthermore, we hypothesize that opaque cells are dead hypertrophied HSCs. Indeed, when we cultured gated #2 cells, the proportion of opaque hypHSCs increased over the days of culture, while that of intact hypHSCs decreased (see Figure S6). Thus, we refined the sorting strategy and gate #2 was separated according to SSC value leading to gate #2–hi and gate #2–med (Figure 3C). Observation and quantification of intact and opaque hypHSCs was then performed on cells obtained from gate #2–hi and gate #2–med by fluorescent microscopy (Figure 3D).

We established that gate #2‐med contained a majority of intact hypHSCs with 56% ± 23% (*n* = 4) against 41% ± 23%, (*n* = 4) of opaque cells. In contrast, the proportion of cells from gate #2‐hi was distributed such that 70% ± 12% (*n* = 4) were opaque cells and 24% ± 10% (*n* = 4) were intact hypHSCs (Figure 3E). Although the presence of these “opaque” cells in the cell preparation obtained after sorting is inevitable, we reduced their proportion as much as possible by selecting only gate #2‐med. This more precise selection made it possible to limit the introduction of excessive bias for subsequent analyses.

Therefore, for further experiments, the final gating strategy chosen for hypHSC sorting was the one corresponding to gate #2‐med (Figure 3C).

In parallel, qHSCs were sorted from a cell preparation derived from SD‐liver using the same cell sorter and following a succession of sorting gates similar to those of hypHSCs, but with a final selection performed on the basis of SSC‐log/blue fluorescence (λ_exc_ = 405 nm; λ_em, max_ = 455 nm) as previously reported in the literature [17, 18].

#### 3.2.2 Cell Characterization After the Sorting Step

At the end of the sorting step, 2.4 ± 1.1 × 10^5^ (*n* = 30) hypHSCs were recovered from CDAHFD‐mouse liver (9–15 weeks of diet) versus 4.4 ± 3.0 × 10^5^ (*n* = 13) cells from a SD‐mouse liver (5 to 10‐month‐old) (see Table 1). No significant difference was observed in the number of cells recovered depending on the diet time (9–19 weeks—see Figure S7). The sorting step recovered approximately 3.5% ± 1.1% of hypHSCs from the total CDAHFD‐cell preparation obtained after density gradient.

**FIGURE 4:**
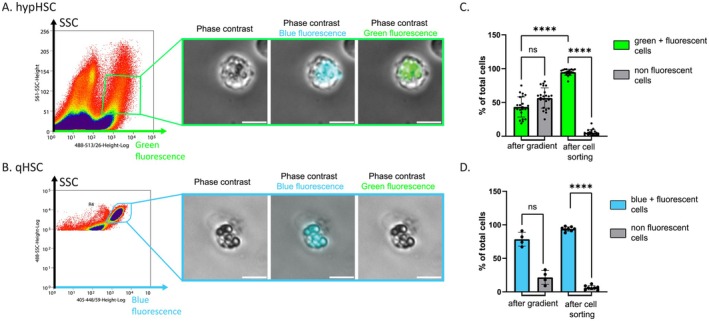
Morphology and characterization of sorted hypHSC and qHSC cell preparations. Representative dot plots with the gates used for sorting (A) hypHSCs (top panel—SSC/green fluorescence) and (B) qHSCs (bottom panel—SSC/blue fluorescence) and corresponding representative microscopy images of sorted hypHSCs and qHSCs. Images of microscopy performed under phase contrast illumination, and merge with green (λ_exc_ = 488 nm, λ_em_ = 500–550 nm) or blue fluorescence (λ_exc_ = 365 nm λ_em_ > 390 nm). Scale bar: 10 μm. (C) Percentage of green fluorescent cells (hypHSCs) and non‐fluorescent cells in preparations obtained after density gradient and after sorting from 9 to 15 weeks CDAHFD‐mice liver. Means ± standard deviation are shown. (after gradient, *n* =23; after sorting, *n* = 19) Kruskall–Wallis test. (D) Percentage of blue fluorescent cells (qHSCs) and non‐fluorescent cells in preparations obtained after density gradient and after sorting from SD‐mice liver. Means ± standard deviation are shown. (after gradient, *n* = 4; after sorting, *n* = 8) One‐way ANOVA test. *ns p* > 0.05; *p ≤ 0.05; **p ≤ 0.01, ***p ≤ 0.001, ****p ≤ 0.0001.

The hypHSC‐sorted preparations were very pure since 94.8% ± 4.5% of the cells were hypHSCs with 22% of mortality (Table 1). Considering that after density gradient the contamination by non‐HSC cells was greater than 50%, cell sorting allowed the elimination of undesirable cells and represents a fundamental step for the further study of hypHSCs (Figure 4C). In comparison, equivalent results in terms of purity and mortality were obtained for the preparations of qHSCs from SD‐mouse liver, underlining that the sorting step is also important for qHSC study (Figure 4D). Importantly, the total duration of purification (dissociation followed by density gradient) and sorting had an effect on the viability of the hypHSCs obtained, as a 2‐day purification increased significantly the percentage of hypHSC mortality (See Figure S7). Following cell sorting, we showed that green and blue fluorescence were indeed associated with well‐defined droplets in hypHSCs, just as blue fluorescence was associated with retinoid droplets in qHSCs (Figure 4A,B).

#### 3.2.3 Expression of Stellate Cell Protein Markers in Sorted hypHSCs

**FIGURE 5:**
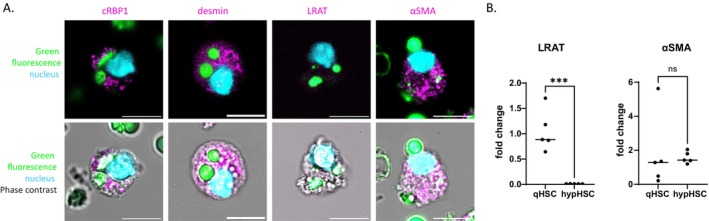
Analysis of stellate cell marker expression in sorted hypHSCs. (A) Representative images of cRBP1, desmin, LRAT and αSMA immunostaining (λ_exc_ = 638 nm, λ_em_ = 650–690 nm, magenta) merged with “green” retinoid fluorescence (λ_exc_ = 488 nm, λ_em_ = 505–535 nm, green), nucleus (DAPI, λ_exc_ = 405 nm, λ_em_ = 425–465 nm, cyan) (top panels) and phase contrast illumination signal (bottom panels) acquired by confocal microscopy. Scale bar 10 μm. (B) Relative expression of LRAT and αSMA in hypHSC vs. qHSC analyzed by qPCR (statistical tests: For LRAT *n* = 5, Unpaired *t*‐test, for αSMA: *N* = 5 Mann–whitney test, ns *p* > 0.05; *p ≤ 0.05; **p ≤ 0.01, ***p ≤ 0.001, ****p ≤ 0.0001).

Thanks to cell sorting, highly pure hypHSC and qHSC preparations were obtained and allowed us to examine the expression of stellate cell markers at the protein level by immunolabeling. Immunofluorescence study of sorted hypHSCs showed that they express desmin, cRBP1 and αSMA confirming what we previously showed by immunohistochemistry on CDAHFD‐liver slices [10] (Figure 5Aand Figure S8 immunolabeling of primary qHSCs from SD mouse liver as a reference). According to qPCR results, the level of αSMA expression in hypHSCs was equivalent to that of qHSCs (Figure 5B). Surprisingly, LRAT immunolabeling was low to not detectable, suggesting that hypHSCs have low LRAT expression that was confirmed by qPCR analysis (Figure 5B).

Thus, we succeeded in purifying the hypHSC we previously identified by fluorescence microscopy on CDAHFD‐mouse liver slice and human fibrotic liver biopsies from CDAHFD‐mouse liver.

### 3.3 Transcriptomic Analysis of hypHSC versus qHSC

As sorted hypHSCs and qHSCs were obtained with 95% purity, this allowed us to further characterize hypHSCs from a molecular point of view. Although no difference was observed at the cellular level depending on CDAHFD time of diet, we decided to analyze sorted hypHSCs from CDAHFD‐mouse liver at restricted duration of 12 to 13 weeks of diet to limit possible molecular variability. Then, NGS sequencing of sorted hypHSCs was performed with sorted qHSCs as reference; bulk analysis allowed us to perform an in‐depth analysis of gene expression.

#### 3.3.1 RNA Extraction From Sorted hypHSCs and qHSCs

The first important criterion before performing RNA sequencing is based on the quality of the extracted RNA, determined by RIN (RNA integrity number), used to produce the sequence library. As the RIN of sorted hypHSCs exhibited significant variability between samples, with RIN values ranging from 3 to 6 (See Figure S7), we therefore optimized the protocol by (i) carrying out the different steps of cell purification (dissociation/gradient/sorting) on the same day, allowing lower cell mortality (See Figure S7), (ii) recovering sorted cells directly in lysis buffer (used for RNA extraction) with an optimal number of cells, (iii) using a RNA extraction kit adapted to low cell number. These optimizations increased the RIN of RNA extracted from sorted hypHSCs with a maximum value of 7.3. Then, for RNAseq analysis of hypHSCs, we selected, among 36 samples obtained from the liver of 19 CDAHFD mice (12–13 weeks of diet), 5 preparations with the highest RINs (mean 6.45). In parallel, 5 preparations of RNA extracted from sorted qHSCs were also selected (mean RIN 7.14 with no difference in mean RIN of selected hypHSC samples).

#### 3.3.2 Bulk RNAseq Analysis of Sorted hypHSCs Versus Sorted qHSCs

Once samples with satisfying RNA quality were selected, non‐strand oriented libraries were prepared as described in the material and method part. Analyses of sequencing data quality, reads repartition (e.g., for potential ribosomal contamination), inner distance size estimation, genebody coverage, strand‐specificity of library were performed using FastQC v0.11.2, Picard‐Tools v1.119, Samtools v1.0, and RSeQC v2.3.9. These first quality controls were assessed using MultiQC including mapping statistics from STAR and read assignments from FeatureCounts.

A total of 16 066 genes were identified on the 10 samples (5 hypHSC, 5 qHSC) during sequencing. Among the genes identified, some genes described as HSC marker genes in the literature [26, 27] were found and expressed by hypHSCs and qHSCs in terms of FPKM (Fragments Per Kilobase per Million mapped fragments), such as *Vimentin*, *Desmin*, *Acta2*, and several collagens (*Col1a1*, *Col1a2*, and *Col3a1*), confirming the stellate cell status of hypHSCs (See Figure S9).

**FIGURE 6:**
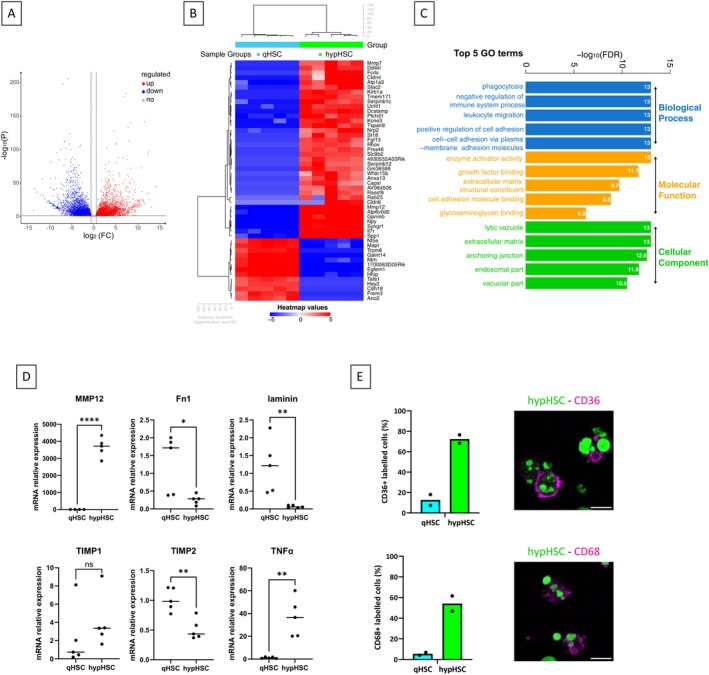
RNAseq analysis of sorted hypHSC and qHSC. (A) Volcano plot depicting differentially expressed genes (DEGs) between hypHSCs and qHSCs. Blue: Downregulated; gray: Unregulated; red: Upregulated. (B) Heatmap and unsupervised hierarchical clustering of the top 50 DEGs, (C) Top 5 gene ontology (GO) analysis terms for biological process, molecular function and cellular component. (D) qPCR analysis of gene expression involved in MEC regulation and inflammation in hypHSCs and qHSCs. Means and distribution of samples according to relative mRNA expression, *n* = 5 independent experiments. *p < 0.05; **p < 0.01; ****p < 0.0001; unpaired t‐test (Mmp12, Fn1, Laminin, Timp2 and Tnfα) or Mann–Whitney test (Timp1). (E) Flow cytometry analysis of CD36 and CD68 labeling on sorted qHSCs or hypHSCs obtained from SD or CDAHFD mice liver respectively. Percentage of labeled cells relative to total cells. *n* = 2 independent experiments. Right panels: Representative images of CD36 and CD68 immunostaining (λ_exc_ = 638 nm, λ_em_ = 650–690 nm, magenta) merged with “green” retinoid fluorescence (λ_exc_ = 488 nm, λ_em_ = 505–535 nm, green) of sorted hypHSCs acquired by confocal microscopy. Scale bar = 10 μm.

Analysis of differentially expressed genes (DEGs) revealed 6926 DEGs between the hypHSC and qHSC populations, with 3369 genes overexpressed by hypHSCs compared with qHSCs and 3557 genes underexpressed by hypHSCs compared with qHSCs (Figure 6A). This result points out a marked difference in gene expression between the two HSC populations.

By identifying the 50 most DEGs, we defined the molecular signature of hypHSCs (Figure 6B). Among the panel of HSC‐historical genes, we observed a strong modification of their expression. Thus, some known gene markers of quiescent HSCs appeared downregulated (such as *Sparc*, *Dcn*, *Csrp2*, *Bambi*…) and others upregulated such as *Adipor1*, *Plin2*, *Vimentin* and *Pparg* (See Table S1). Interestingly, the “gold standard” canonical genes of quiescent HSCs such as *Lrat* (FC 66.45), *Gfap* (FC 43.48) and *Desmin* (FC 20.82) appeared to be down regulated, although the latter was detectable at protein level on sorted hypHSCs (Figure 5A). Moreover, most of the genes historically known as markers of activated HSC did not appear deregulated, such as *Acta2*, collagens (*Col1a1*, *Col1a2*, *Col3a1*), *Notch3* and *Timp1* [3, 4, 28] (See Figure S9 and Table S1). We also examined the expression of specific HSC genes identified in several transcriptomic studies (scRNAseq) [5, 6, 8, 29, 30, 31], such as *Tmem56*, *Colec10*, *Bco1*, *Plvap*, *Vipr1* (see all in Figure S9B) and showed that these genes were expressed in qHSCs and downregulated in hypHSCs, which is to be expected since these markers were identified from quiescent HSCs derived from normal liver tissue and specific to these cells compared to other mesenchymal cells in the liver.

Among the genes upregulated in hypHSCs it is interesting to note the upregulation of several genes involved in fatty acid uptake such as *Cd36*, *Fatp1* and some FABPs (*Fabp5, Fabp4*) as well as *Ldl*, suggesting an upregulation of fatty acid uptake and activation/transport by hypHSCs. *Cd36* upregulation was confirmed at the protein level by flow cytometry analysis with an average of 53% more expression than qHSCs (Figure 6E). However, this seemed not associated with metabolization and storage of fatty acids into triglycerides (TG) as the first step of TG biosynthesis appeared almost inhibited (*Gpat2* down regulated, FC = 228.81) whereas TG catalysis into diacylglycerol (DAG) seemed to be induced with *Pnpla2* (*Atgl*, FC = 2.82) and *Plin2* (FC = 3.43) up regulated in hypHSCs (Table S2).

Interestingly, expression of key enzymes of the retinoid metabolism seemed completely disrupted in hypHSCs. Our data showed that the retinol esterification pathway is almost inhibited in hypHSCs compared to qHSCs, with a marked decrease in *Lrat* expression observed in both RNA‐seq and qPCR data (as shown on Figure 5 of the manuscript). Indeed, two pathways leading to the formation of all‐trans retinol are overexpressed in hypHSCs: the retinyl ester hydrolysis into all‐trans retinol, with overexpression of *Pnpla2*; and the conversion reaction of all‐trans retinal to all‐trans retinol, with overexpression of *Dhrs3* (FC = 2.06) and *Rdh13* (FC = 3.14) (Table S2). Thus, it appears that expression of key enzymes of the retinoid metabolism may be completely unbalanced towards the formation of all‐trans retinol.

Given the large number of deregulated genes, gene ontology (GO) analysis was performed to help study the pathways and functions that are deregulated in hypHSCs without “a priori” assumptions. GO classifies the way in which gene functions can be described into three distinct categories: biological process, molecular function and cellular component (Figure 6C). This analysis showed that hypHSCs interact with the immune system, have down‐regulated cell adhesion properties, exhibited phagocytosis/endocytosis functions (upregulated), and may be involved in regulating of the composition of extracellular matrix.

Concerning the regulation of extracellular matrix composition, *Mmp12* and *Mmp7* appeared to be upregulated and within the first 50 DEGs, while laminin and fibronectin appeared to be downregulated. Upregulation of *Mmp12* expression and downregulation of *laminin, fibronectin*, and *Timp2* expression was confirmed by qPCR (Figure 6D), suggesting a role for hypHSCs in the dysregulation of the synthesis/degradation balance of the surrounding extracellular matrix.

Moreover, some genes involved in inflammation and regulation of immune system processes appeared to be significantly upregulated, such as *Il7r* (in the TOP 50 DEGs) and *Tnfα*, with an average mRNA expression 36.6 times higher than in qHSCs (Figure 6D).

Among genes present in GO terms “negative regulation of immune system” and “leukocyte migration”, *Il7r* (FC 1824; in the TOP 50 DEGs) and *Tnfα*/*Cxcl2* cytokine genes (FC 28.93 and 35.88) appeared to be significantly upregulated. Upregulation of *Tnfα* was confirmed by qPCR with an average mRNA expression 36.6 times higher than in qHSC (Figure 6D). Moreover, MHC (major histocompatibility complex) genes, in particular *Cd74* (FC 5.74) and *Ctss* (FC 60.74), appear among the 100 most expressed genes by hypHSCs in terms of FPKM. This suggests that hypHSCs may have an activity related to inflammation and regulation of immune system process.

Finally, phagocytosis/endocytosis process appeared also upregulated with upregulation of genes such as *Cd36* (phagocytosis/uptake of fatty acids), *Cd68* and *Lamp1* (lysosomal function) as well as *Cd63* (endocytosis). At the protein level, we confirmed the increased expression of Cd36 and Cd68 with respectively 59.7% and 48.7% higher labeling for hypHSCs compared to qHSCs (Figure 6E).

Taken together, these results suggest that hypHSCs cannot be defined as “classical” activated HSCs or quiescent HSCs. They may suggest that hypHSCs exhibit a hybrid profile involved in different signaling pathways such as fibrogenesis, lipid and retinoid metabolism, and interaction with the immune system that does not correspond to the HSC subtypes already described in the context of liver fibrosis.

## 4 Discussion

In this work, we have described the strategy for isolating hypHSCs, a specific population of HSCs associated with hepatic fibrosis. Although the protocol for HSC isolation is widely described for healthy or fibrotic murine liver (mainly CCl_4_ model) [17, 19], there are only a few papers on HSC isolation from fatty and fibrotic liver. To our knowledge, only one study has performed HSCs isolation from a CDAHFD liver, after 3 weeks of diet. In these conditions, the liver does not yet present fibrosis but already steatosis [32]. In the same way, one paper reports the isolation of HSCs from mice fed a “Western diet” for 12 weeks [7] without specifying the influence of steatosis on HSC purification or HSC purity. Thus, the hypHSC isolation protocol we describe here, based on the CDAHFD model and characterized in a large number of mice (*n* = 19), can be applied to studies on HSC in a context of metabolic hepatic fibrosis. Our study also highlights the importance of cell sorting after the density gradient, as FACS yielded an ultra‐pure hypHSC fraction (95%) from an enriched hypHSC fraction (43%) after the density gradient.

Our results report an augmentation of bacterial load in the liver of CDAHFD‐mice compared to healthy mice. These observations are consistent with literature data describing a link between MALFD/MASH and disruption of the intestinal microbiota. For example, mice fed a high‐fat (HF) or choline‐ and methionine‐deficient (MCD) diet have an abnormal composition of the intestinal microbiota, leading to a decrease in the thickness of the mucous layer of the intestinal barrier and a redistribution of tight junctions forming proteins [23, 33]. Increased intestinal permeability and impaired tight junctions have also been observed in patients with MAFLD [34]. The onset of intestinal bacteria in the liver causes hepatic inflammation as bacteria and their metabolites constitute PAMPs (Pathogen‐Associated Molecular Pattern) that can bind to TLR‐4 and TLR‐9 receptors on Kupffer cells [35, 36].

HSCs are known to play a role in the defense against bacterial infection, notably through the secretion of cytokines and chemokines that induce neutrophil recruitment and interaction with immune cells [37, 38]. Gene ontology analysis of our transcriptomic data revealed 2 top GO terms to be “negative regulation of immune system” and “leukocyte migration” notably with upregulation of the *Tnfα/Cxcl2* cytokine genes and MHC genes such as *Cd74* and *Ctss*, which may be associated with antigen presentation and immune cell recruitment for the response to bacterial infection [39, 40].

Thus, our data showed that the purification of HSCs from mice subjected to a CDAHF diet requires precautions such as adding antibiotics to the different solutions used. In addition, these results indicated that the use of the CDAHFD model could be interesting to study microbiota and bacterial translocation in the context of metabolic liver disease.

In this paper, we showed that the characteristic fluorescence properties of hypHSCs in the green range allowed their specific detection during isolation from an enriched fraction of HSCs obtained after density gradient. Using the fluorescence properties of retinoid droplets in the blue range is a well‐known and widely used strategy for the purification of quiescent HSCs [17, 18, 19] but to our knowledge, our study is the first that took advantage of distinct intrinsic fluorescent properties to isolate a particular HSC population in a fibrotic context. The different fluorescence properties between hypHSC and qHSC retinoid droplets may be due to a different composition in terms of triglycerides or fatty acids or even a difference in lipid bilayer morphology, as it has been demonstrated to occur during HSC activation [41, 42, 43]. Moreover, an increase of free cholesterol content in HSC has been shown in murine models of nonalcoholic steatohepatitis [44]. Then, further study on the content of hypHSC lipid droplets could be of interest to understand the dynamic of retinoid droplets that occurs upon HSC hypertrophy in the context of hepatic fibrosis and for such a study, isolated hypHSCs are necessary.

While these specific properties allowed us to distinguish hypHSCs from other cells, they seemed to add difficulty for their characterization based on fluorescence analysis methods. Indeed, this fluorescence broad spectrum may induce a high “noise” signal that has to be considered and could require spectral acquisition and compensation analysis for further study (See Figure S10). All steps of the process were monitored by cell microscopy observation to ensure that they contained intact and fluorescent droplets. Thus, careful microscopy observation of sorted cells allowed us to highlight the presence of hypHSCs with high fluorescent signal and no longer well‐defined fluorescent droplets that we called “opaque” hypHSCs. Some studies showed that cell sorting may induce cell stress and impact cell metabolism suggesting that isolation method should be determined carefully to limit the inaccuracy of further analysis [45, 46]. As opaque hypHSCs did not reflect the phenotype in which we had previously identified them in CDAHFD‐mice liver sections and human biopsies, and as their proportion increased with the number of days in culture, we hypothesized that “opaque” hypHSCs corresponded to cells damaged by the FACS process and defined a sorting strategy that minimized the presence of these cells as much as possible. In addition, we are also working to determine optimal culture conditions for sorted hypHSCs and plan to perform precision‐cut liver slice (PCLS) cultures for future functional experiments [47, 48].

Bulk RNAseq analysis of hypHSCs allowed us to perform in‐depth analysis of gene expression and to determine a difference of gene expression for 6926 genes between hypHSCs and qHSCs. Our results showed that hypHSCs resemble neither qHSCs nor activated HSCs. It is now well known that the paradigm of “quiescent” or “activated” cellular states for HSCs no longer fully reflects the heterogeneity of HSC subtypes, as scRNAseq studies shed light on the dynamics of HSC‐myofibroblast transition in response to liver injury. However, none of these studies identified hypHSCs, possibly due to the strategy chosen to isolate HSCs, based on transgenic mice expressing a reporter gene under a HSC‐specific promoter such as that of *Lrat* or *Pdgfrβ* [5, 49]. Such a strategy, rather elegant for the isolation of cells, is likely to induce a bias in the study since it assumes that HSCs are characterized by a unique marker, the gene associated with the promoter, contrary to the strategy we used here, based on the specific and endogenous fluorescence of hypHSCs in the green range.

However, some scRNAseq studies based on a non‐parenchymal hepatic fraction (i.e., after gradient) or on sorted HSCs based on ‘blue’ fluorescence properties in several fibrotic contexts (CCl_4_, MASH or cholestatic murine model) should have recovered hypHSCs simultaneously with other HSCs subtypes. For example, we found that hypHSCs could correspond to the cluster of activated HSCs highly expressing genes associated with leukocyte activation and immune regulation such as *Slpi*, *Cd74* and *Dmkn* described in Krenkel'study [6]. By comparing the genes expressed by hypHSCs, we hypothesized that they could also correspond to cluster #7 of Rosenthal'study which is part of the 3 HSC clusters not described in details [7]. Indeed, our transcriptomic data showed that hypHSCs underexpressed certain typical HSC genes such as *Lrat*, *Pdgfr* or *Desmin*, compared to qHSCs.

Analysis of transcriptomic data suggested that hypHSCs exhibit strong fatty acid uptake and lipolysis activity, and particularly a high activity of hydrolysis of retinyl esters into retinol. Retinyl ester breakdown is a known feature for activated HSC, jointly with profibrogenic gene expression and in association with the loss of retinoid droplets [50, 51]. However, in hypHSCs, we observed an exacerbation of retinoid droplets which, contrary to what might be expected, may not be due to excessive storage of retinyl esters in lipid droplets. Indeed, our results showed a strong down regulation of LRAT expression in hypHSCs. On the contrary, our data suggested an increase of retinyl ester hydrolysis reaction into all‐*trans‐*retinol in hypHSCs. The data do not allow us to draw definitive conclusions about the use of retinol and fatty acids, as they may be implicated in several processes, as for example all‐*trans*‐retinoic acid and certain fatty acids play a role in transcriptional activity mediated by RAR, RXR and PPARγ [52]. Further studies, particularly in lipidomics, would be necessary to understand the lipid metabolism pathways involved in hypHSCs.

Thus, our data suggest that hypHSCs have a hybrid profile involved in several processes with expression markers specific to phagocytosis and inflammation, exhibiting a deregulation of lipid metabolism while expressing some markers of extracellular matrix remodeling.

Then, we demonstrated here that by adopting a “top‐down” approach (from the specific phenotypic characteristics of hypHSCs down to their gene expression), we were able to isolate and characterize the hypHSC molecular signature that differs from already described HSC subtypes in the fibrosis context.

Based on this study, further investigations may help elucidate hypHSCs status among the different HSC subpopulations that coexist during hepatic fibrosis and better understand their role in the mechanisms of fibrosis.

## Author Contributions

Marion Heckmann, Nour‐El‐Houda Djerir, Keola Greliche and Céline Hoffmann performed the experiments and analyzed the data, Pierre‐Henri Commere provide resources for FACS, advices and performed the sorting, Julien Fernandes provided microscopy resources, advices and performed microscopy acquisitions, Guillaume Sarrabayrouse performed experiments and analysis on mice microbiota, Marion Heckmann, Pascal Bigey, Bernard Hainque, Virginie Escriou and Céline Hoffmann performed formal analysis on RNAseq, Virginie Escriou and Céline Hoffmann designed the methodology, supervised the project and wrote the manuscript with input from all authors. All authors had access to the study data and had reviewed and approved the final manuscript.

## Funding

This work was supported by the Agence Nationale de la Recherche (ANR, ANR Fibrother ANR‐18‐CE18‐0005‐01 to Céline Hoffmann) and has received support under the program “Investissement d'Avenir” launched by the French Government and implemented by ANR, with the reference ANR‐18‐IdEx‐0001 as part of its program Emergence (Free‐hypHSC project to Céline Hoffmann and Julien Fernandes). The UtechS PBI (Julien Fernandes) is part of the France BioImaging infrastructure supported by the French National Research Agency (ANR‐10‐INSB‐04‐01, “Investments for the future”).

## Disclosure

Preprint Server: This work is available on bioRXiv as a pre‐print with the following DOI: 10.1101/2025.05.19.654829.

## Conflicts of Interest

The authors declare no conflicts of interest.

## Supporting information


**Figure S1:** Kinetic of fibrosis and HSC hypertrophy progression as a function of CDAHFD time. (A) Histological colorations (top: Hematoxylin/Eosin, low: Sirius Red). (B) HSC hypertrophy (blue dots) and fibrosis (red triangles) scores as a function of CDAHFD time. (*n* = 10–14 for each time point, means ± standard deviation). (data from Hoffmann et al., Scientific Reports, 2020) (C) Examples of HSC hypertrophy (pointed by an arrow) on CDAHFD‐mouse liver observable on histological sections stained with H&E, Sirius Red and on paraffin slices by fluorescence microscopy. Scale bar: 50μm.
**Figure S2:** Bacterial contamination of cell preparations obtained from CDAHFD mice. (A‐B) Cell preparations from CDAHFD mouse without (A) and with (B) antibiotics during HSC purification. Cell preparation with antibiotics contains less bacteria than cell preparation without antibiotics in buffers. (C) Example of cell preparation from SD mouse without antibiotic. (D) Evaluation of fecal bacterial load as a function of diet time. Representation of the number of copies of the gene encoding 16S rRNA obtained after amplification by qPCR. Statistics: *n* = 5 for each SD diet time, *n* = 8 for 3, 6 and 9 weeks of CDAHF diet and *n* = 4 for 12 weeks of CDAHF diet, mean ± SD, Mann–Whitney test, *p < 0.05. (E) Evaluation of liver bacterial load as a function of diet. Graph representative of the number of copies of the gene encoding 16S rRNA obtained after amplification by qPCR for 3 liver samples from SD‐fed mice and 3 CDAHFD‐fedmice (top). Image of genomic gel of corresponding samples after amplification of V4 region of the 16S rRNA gene by standard PCR (bottom).
**Figure S3:** Localization of cells obtainedfrom CDAHFD‐and SD‐mouse liver after density gradient on FSC/SSC dot plot. (A) Dot plot of cell preparations analyzed as a function of FSC and SSC. (B) Dot plot of the same cell preparations without labeling, analyzed according to green fluorescence (λexc= 488 nm and λem= 525 nm) and yellow fluorescence (λexc= 488 nm and λem= 583 nm). (C) Dot plot of the cell preparations labeled with SYTO16. The points corresponding to the positive SYTO16 labelling are represented in green. (D) The points corresponding to the syto16‐labelled events, therefore to intact cells are represented in green on the FSC‐SSC dotplot.
**Figure S4:** Flow cytometry analysis of liver cell markers on a cell preparation obtained after density gradient from CDAHFD mouse liver. Representative dot plots used for Figure 2C. (A) Intact cells (green) are identified by Syto 16 labeling as shown in Figure S3. (B‐G) Representative dot plots of Red2 fluorescence (autofluorescence, λexc = 640 nm and λem = 661 nm) as a function of Green fluorescence (marker labeling, λexc = 488 nm and λem = 525 nm), for different markers. (B) The autofluorescence signal in Red2 laser emitted by the cells, mainly by the hypHSCs, interferes with the evaluation of the labeling when the data are represented in the form of a histogram (see Figure S10), which explains the choice of a dot plot representation. (C–E) Representative dot‐plots of ASGPR (hepatocytes), F4/80 (macrophages) and CD31 (endothelial cells) labeling on unpermeabilized cells as these are extracellular surface markers; (F‐G) Representative dot‐plots of desmin and cRBP1 labeling on permeabilized cells as these are typical intracellular markers of HSCs. The percentage of positive cells for each marker is indicated in red on the dot plot. The percentage of cells nonspecifically labeled with the secondary antibody conjugated to Alexa 647 alone (leftmost dot plot for each marker) is approximately 4%–5% for non‐permeabilized cells (C‐E, ASGPR, CD31, and F4/80 markers) and negligible (< 1%) for permeabilized cells (F‐G, desmin and cRBP1 markers).
**Figure S5:** SSC scale adjustment strategy for precise selection of hypHSC on SSC/green fluorescence dot plot.
(A) log‐SSC/log‐FSC dot plot allowing to determine the gate for cells, (B) doublet cell elimination, (C) dot plot for log‐SSC on 488 laser/log‐green fluorescence (488 laser), (D) dot plot for log‐SSC on 561 laser/log‐green fluorescence (488 laser) and example of gating (red circle) to highly green fluorescent cells. (E) application of the SSC‐561 scale in linear mode on dot plot for SSC‐561/log‐green fluorescence showing position of selected cells on E on highly fluorescent and highly granular cells, and highlighting that using log‐SSC mode led to miss low SSC‐high fluorescent cells (red dotted area).
**Figure S6:** Evolution of hypHSC phenotype as a function of culture time. (A) Phase contrast and green fluorescence (λexc = 488 nm, λem = 500–550 nm) microscopy images at t = 0, 3 and 7 days after sorting. Bars = 50 μm. (B) Quantification of opaque and intact hypHSCs by microscopy at a function of time (Mann–Whitney test, *n* = 3‐4 independent experiments). (C) Phase contrast and green fluorescence (λexc = 488 nm, λem = 500–550 nm) microscopy images of different phenotypes of hypHSCs according to the state of fluorescent droplets.
**Figure S7:** (A) Number of recovered cells after FACS as a function of CDAHFD time. Statistics: for 11 weeks *n* = 1; for 9, 14 and 15 weeks: *n* = 2, for 10 and 13 weeks and for 12 weeks: *n* = 8, mean ± standard deviation, Kruskall–Wallis test not significant. (B) Mortality of hypHSCs collected after sorting according to the duration of purification (1 or 2 days). Statistics: *n* = 13 CDAHFD mice for both conditions, mean ± SD, **p.
**Figure S8:** Immunolabeling of stellate cell markers on primary murine HSC purified from SD mouse liver. Purified qHSCs from SD mouse liver were seeded in microwells mounted on a polymer coverslip (Ibidi) at a density ranging from 6000 to 10000 cells/cm² and left to adhere for 16 h at 37°C/5% CO2 and fixed with PFA 4% for immunolabeling as described in material and methods. Representative images of cRBP1, desmin, αSMA and LRAT immunostaining (λexc = 638 nm, λem = 650–700 nm, magenta) merged with retinoid autofluorescence in the blue range (λexc = 405 nm, λem = 415–490 nm, cyan) (top panels) and actin stained with fluorescent phalloidin, to visualize cells (λexc = 552 nm, λem = 560–630 nm, red) (bottompanels) acquired by confocal microscopy. Scale bar 10 μm.
**Figure S9:** Analysis of the expression of some HSC canonical (A) and specific marker (B) genes by quiescent and hypertrophied HSCs. FPKM (Fragments Per Kilobase of transcription per Million mapped reads) mean of 5 samples for each condition. (A) Acta2= α‐SMA; Col1a1, Col1a2 and Col3a1 = collagens 1a1, 1a2 and 3a1; Vim=vimentin; Lgals1 = galectin‐1; Des = desmin; Lrat = lecithin retinol acyltransferase; Pdgfrb = PDGFR‐β; Dcn = decorin; Fn1 = Fibronectin; Sparc = osteonectin; Csrp2 = cysteine‐ and glycine‐rich protein. (B) Tmem56/Tlcd4 (TLC domain containing 4), Colec10 = Collectin subfamily member, Bco1 = betacarotene oxygenase 1, Plvap = plasmalemmavesicle associated protein, Fcna = ficolin a, Pth1r = parathyroid hormone 1 receptor, Angptl6 = angiopoietin like 6, Lrat = lecithin retinol acyltransferase, Reln = Reelin, Vipr1 = Vasoactive intestinal peptide receptor 1, Ngfr = Nerve Growth Factor receptor.
**Figure S10:** Representative histograms from flow cytometry analysis of sorted hypertrophied and quiescent HSC. Hypertrophied (hypHSC) and quiescent (qHSC) hepatic stellate cells were analyzed by flow cytometry and Red2 fluorescence signals were recorded and displayed as histograms. (A) unstained cells. (B) unstained cells (up) and cells stained with Alexa647‐labeled anti‐desmin antibody (down). HypHSC show a very broad fluorescence signal, much more wide ranging than that of qHSCs. When these two cell types are analyzed by flow cytometry, e.g., with excitation at 647 nm, qHSCs show a classic peak in the low range of fluorescence intensity, whereas hypHSCs show a much wider distribution of cells as a function of fluorescence intensity, with a heterogeneous profile. When both cell types are incubated with an anti‐desmin antibody coupled to the Alexa 647 fluorophore, the qHSC peak shifts towards higher intensities, univocally demonstrating labeling of all cells. For hypHSCs, the shape of the distribution profile changes, but the range of intensities remains unchanged, making it impossible to demonstrate specific labeling.
**Table S1:** Regulation of HSC specific genes. Table of HSC specific genes with regulation in hypHSC compared to qHSC, value of fold‐change, p‐value and corresponding expression value (FPKM) and rank for hypHSC and qHSC. Data normalized using RPKM/FPKM method are used to compare expression between genes within a sample, while fold change between samples (Differential expression analysis) used DEseq2 method of normalization. NR = unregulated, up = up‐regulated (pink), down = down‐regulated (green).
**Table S2:** Regulation of lipid metabolism pathways in hyp HSC versus qHSC. Table genes with regulation in hypHSC compared to qHSC, value off old‐change, p‐value and corresponding expression value (FPKM) for hypHSC and qHSC. Data normalized using RPKM/FPKM method are used to compare expression between genes within a sample, while fold change between samples (Differential expression analysis) used DE seq2 method of normalization. NR = unregulated, up = up‐regulated (pink), down = down‐regulated (green).
**Table S3:** Primer sequences used for qPCR analysis of gene expression.

## Data Availability

The RNA‐sequencing data that support the findings of this study have been deposited in GEO database under the accession number GSE294603. All other relevant data are within the paper and its Supporting Information files.
