# Supplementary material for: Purification and Transcriptomic Characterization of Hypertrophied Hepatic Stellate Cells From CDAHFD Mouse Liver
Source: FASEB J. 2026 Apr 23;40(8):e71754. doi: 10.1096/fj.202502655RR (PMC13104797; doi:10.1096/fj.202502655RR)
Supplement: Supplementary file 1 — Figure S1: Kinetic of fibrosis and HSC hypertrophy progression as a function of CDAHFD time. (A) Histological colorations (top: Hematoxylin/Eosin, low: Sirius Red). (B) HSC hypertrophy (blue dots) and fibrosis (red triangles) scores as a function of CDAHFD time. (n = 10–14 for each time point, means ± standard deviation). (data from Hoffmann et al., Scientific Reports, 2020) (C) Examples of HSC hypertrophy (pointed by an arrow) on CDAHFD‐mouse liver observable on histological sections stained with H&E, Sirius Red and on paraffin slices by fluorescence microscopy. Scale bar: 50μm. Figure S2: Bacterial contamination of cell preparations obtained from CDAHFD mice. (A‐B) Cell preparations from CDAHFD mouse without (A) and with (B) antibiotics during HSC purification. Cell preparation with antibiotics contains less bacteria than cell preparation without antibiotics in buffers. (C) Example of cell preparation from SD mouse without antibiotic. (D) Evaluation of fecal bacterial load as a function of diet time. Representation of the number of copies of the gene encoding 16S rRNA obtained after amplification by qPCR. Statistics: n = 5 for each SD diet time, n = 8 for 3, 6 and 9 weeks of CDAHF diet and n = 4 for 12 weeks of CDAHF diet, mean ± SD, Mann–Whitney test, *p < 0.05. (E) Evaluation of liver bacterial load as a function of diet. Graph representative of the number of copies of the gene encoding 16S rRNA obtained after amplification by qPCR for 3 liver samples from SD‐fed mice and 3 CDAHFD‐fedmice (top). Image of genomic gel of corresponding samples after amplification of V4 region of the 16S rRNA gene by standard PCR (bottom). Figure S3: Localization of cells obtainedfrom CDAHFD‐and SD‐mouse liver after density gradient on FSC/SSC dot plot. (A) Dot plot of cell preparations analyzed as a function of FSC and SSC. (B) Dot plot of the same cell preparations without labeling, analyzed according to green fluorescence (λexc= 488 nm and λem= 525 nm) and yellow fluorescence (λe [file FSB2-40-e71754-s001.pdf]

Supplemental information

Purification and transcriptomic characterization of hypertrophied hepatic stellate cells from CDAHFD mouse liver

Authors

Marion Heckmann, Nour-El-Houda Djerir, Keola Greliche, Pierre-Henri Commere, Julien Fernandes, Guillaume Sarrabayrouse, Bernard Hainque, Pascal Bigey, Virginie Escriou, Céline Hoffmann*

Supplemental Figures – Figure S1

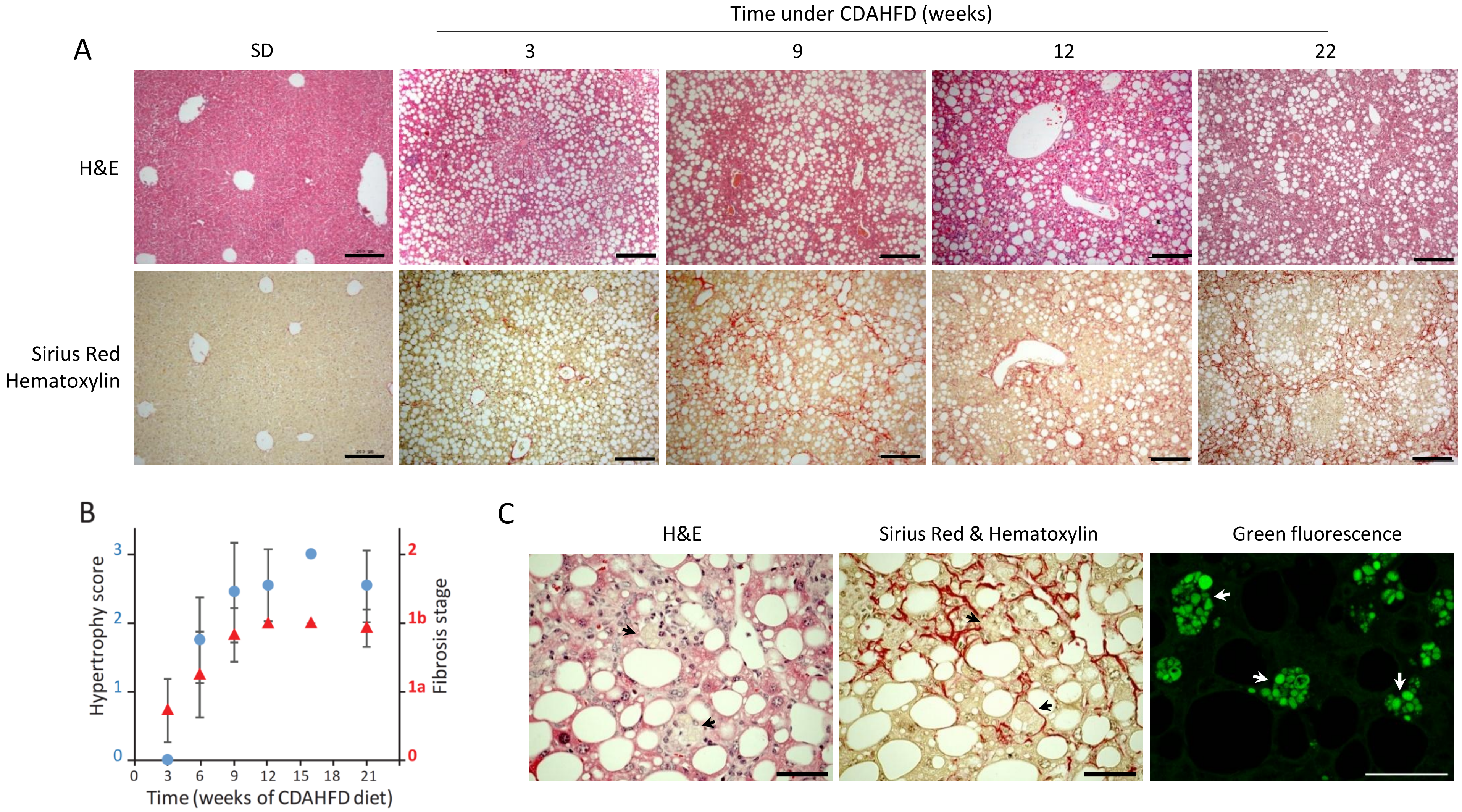

Figure S1: Kinetic of fibrosis and HSC hypertrophy progression as a function of CDAHFD time. A) Histological colorations (top: Hematoxylin/Eosin, low: Sirius Red). B) HSC hypertrophy (blue dots) and fibrosis (red triangles) scores as a function of CDAHFD time. (n = 10–14 for each time point, means ± standard deviation). (data from Hoffmann *et al.*, Scientific Reports, 2020) C) Examples of HSC hypertrophy (pointed by an arrow) on CDAHFD-mouse liver observable on histological sections stained with H&E, Sirius Red and on paraffin slices by fluorescence microscopy. Scale bar : 50µm

Supplemental Figures – Figure S2

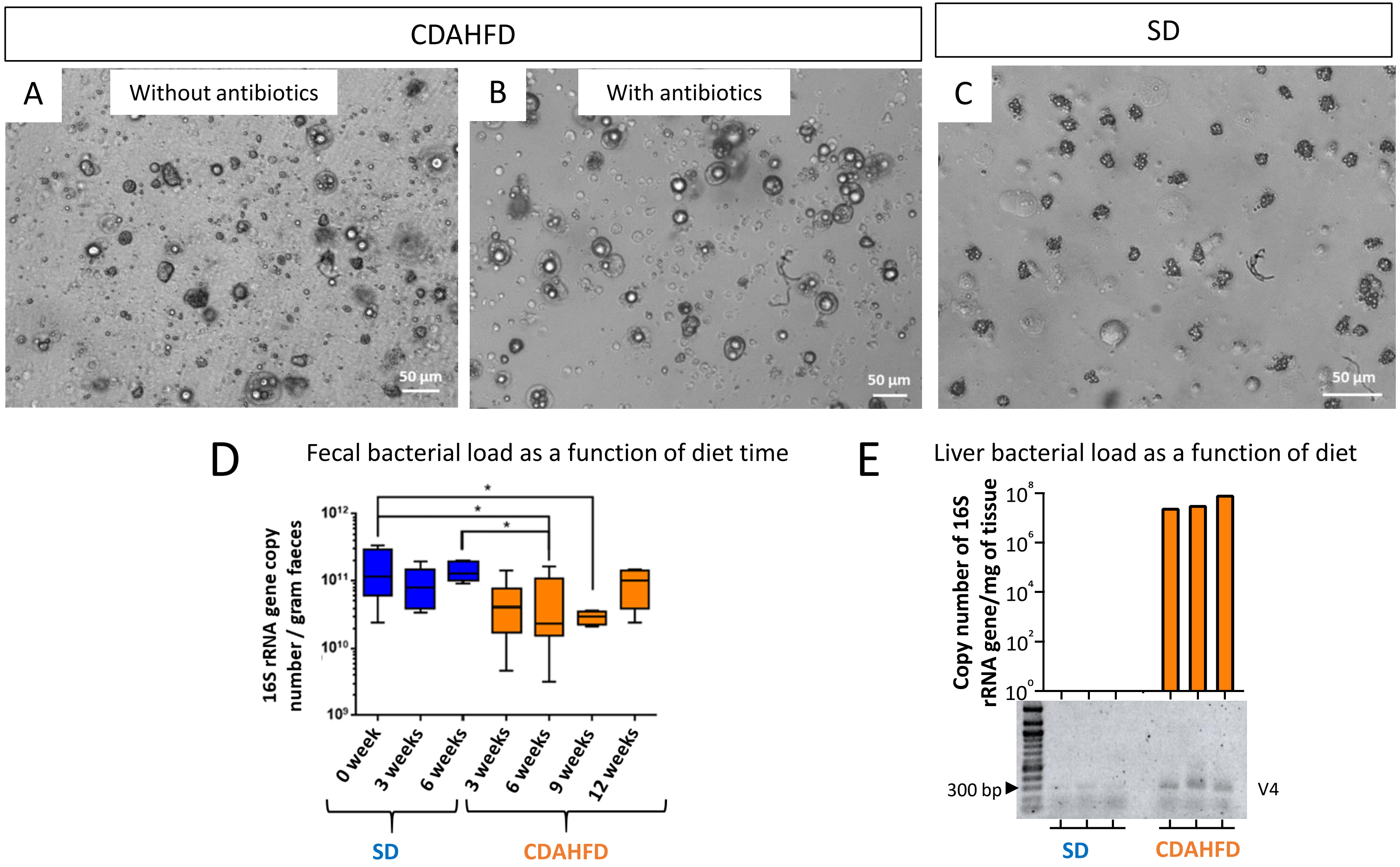

Figure S2: Bacterial contamination of cell preparations obtained from CDAHFD mice. A-B) Cell preparations from CDAHFD mouse without (A) and with (B) antibiotics during HSC purification. Cell preparation with antibiotics contains less bacteria than cell preparation without antibiotics in buffers. **C) Example of cell preparation from SD mouse** without antibiotic. **D) Evaluation of fecal bacterial load as a function of diet time.** Representation of the number of copies of the gene encoding 16S rRNA obtained after amplification by qPCR. Statistics: n = 5 for each SD diet time, n = 8 for 3, 6 and 9 weeks of CDAHF diet and n = 4 for 12 weeks of CDAHF diet, mean ± SD, Mann-Whitney test, *$p<0.05$. **E) Evaluation of liver bacterial load as a function of diet.** Graph representative of the number of copies of the gene encoding 16S rRNA obtained after amplification by qPCR for 3 liver samples from SD-fed mice and 3 CDAHFD-fed mice (top). Image of genomic gel of corresponding samples after amplification of V4 region of the 16S rRNA gene by standard PCR (bottom).

Supplemental Figures – Figure S3

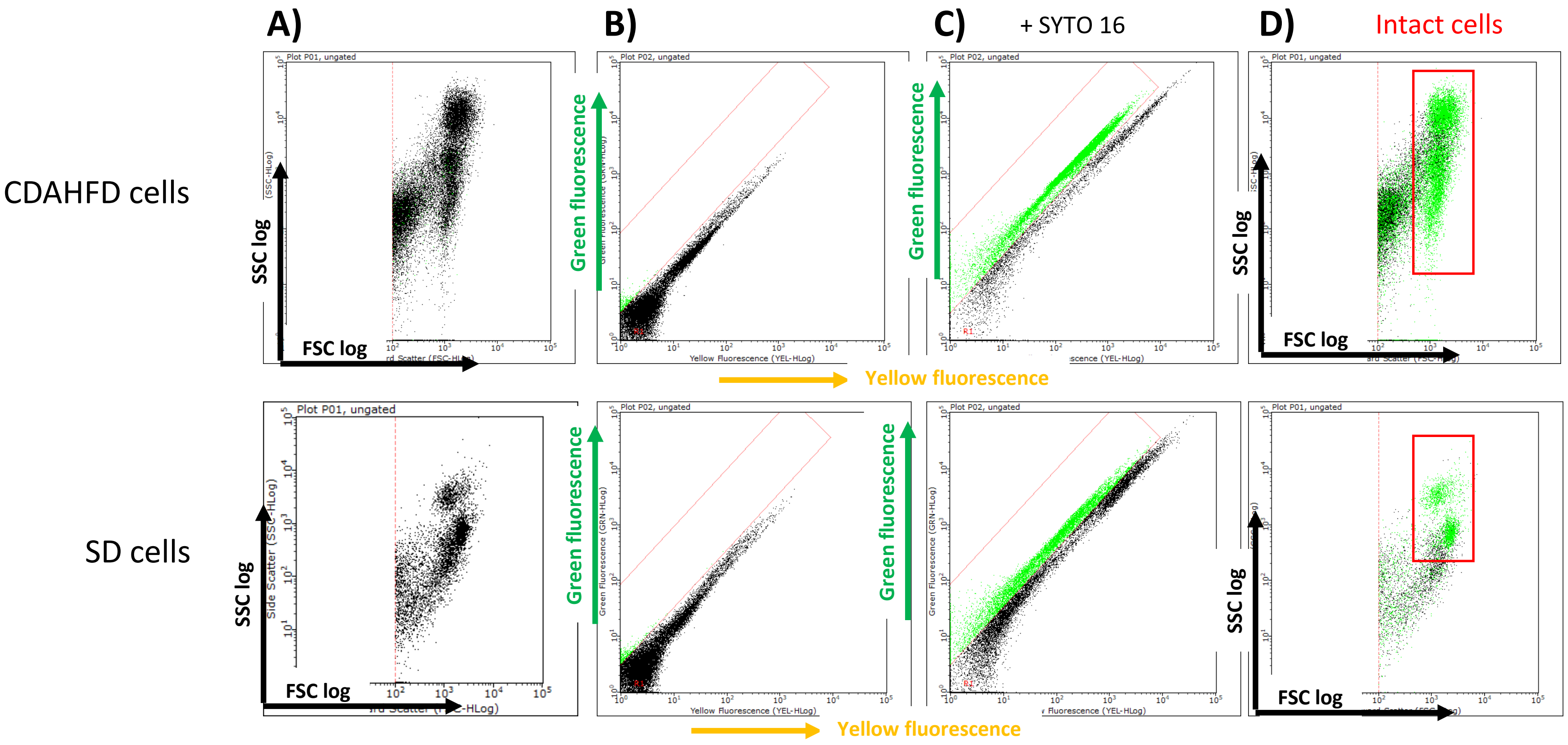

Figure S3 : Localization of cells obtained from CDAHFD- and SD-mouse liver after density gradient on FSC/SSC dot plot. A) Dot plot of cell preparations analyzed as a function of FSC and SSC. B) Dot plot of the same cell preparations without labeling, analyzed according to green fluorescence (λexc = 488 nm and λem = 525 nm) and yellow fluorescence (λexc = 488 nm and λem = 583 nm). C) Dot plot of the cell preparations labeled with SYTO16. The points corresponding to the positive SYTO16 labelling are represented in green. D) The points corresponding to the syto16-labelled events, therefore to intact cells are represented in green on the FSC-SSC dot plot.

Supplemental Figures – Figure S4

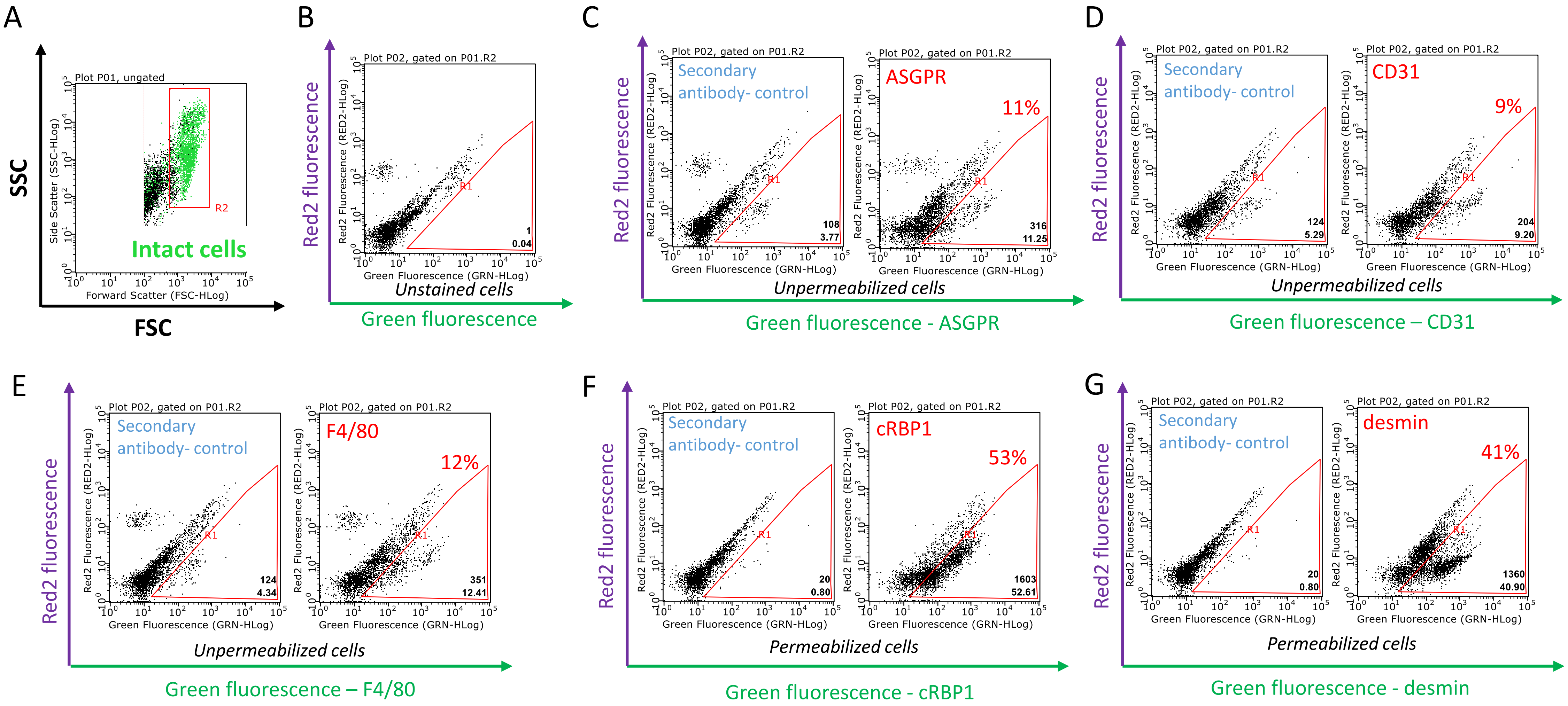

Figure S4 – Flow cytometry analysis of liver cell markers on a cell preparation obtained after density gradient from CDAHFD mouse liver. Representative dot plots used for figure 2.C. (A) Intact cells (green) are identified by Syto 16 labeling as shown in Figure S3. (B-G) Representative dot plots of Red2 fluorescence (autofluorescence, λ_{exc} = 640 nm and λ_{em} = 661 nm) as a function of Green fluorescence (marker labeling, λ_{exc} = 488 nm and λ_{em} = 525 nm), for different markers. B)The autofluorescence signal in Red2 laser emitted by the cells, mainly by the hypHSCs, interferes with the evaluation of the labeling when the data are represented in the form of a histogram (see figure S7), which explains the choice of a dot plot representation. (C-E) Representative dot-plots of ASGPR (hepatocytes), F4/80 (macrophages) and CD31 (endothelial cells) labeling on unpermeabilized cells as these are extracellular surface markers; (F-G) Representative dot-plots of desmin and cRBP1 labeling on permeabilized cells as these are typical intracellular markers of HSCs. The percentage of positive cells for each marker is indicated in red on the dot plot. The percentage of cells nonspecifically labeled with the secondary antibody conjugated to Alexa 647 alone (leftmost dot plot for each marker) is approximately 4–5% for non-permeabilized cells (C-E, ASGPR, CD31, and F4/80 markers) and negligible (<1%) for permeabilized cells (F-G, desmin and cRBP1 markers).

Supplementary figure S5

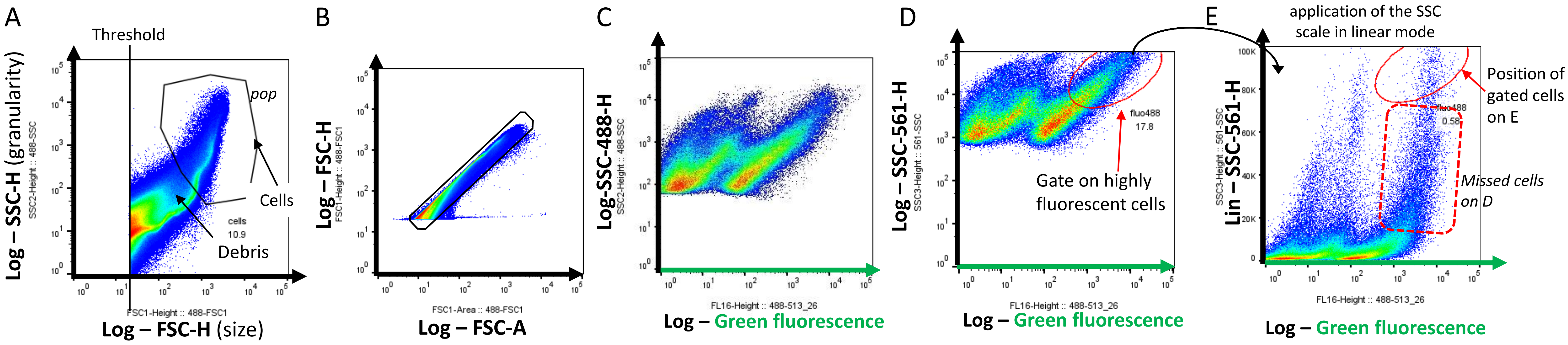

Supplementary figure S5. SSC scale adjustment strategy for precise selection of hypHSC on SSC/green fluorescence dot plot.
A) log-SSC/log-FSC dot plot allowing to determine the gate for cells, B) doublet cell elimination, C) dot plot for log-SSC on 488 laser /log-green fluorescence (488 laser), D) dot plot for log-SSC on 561 laser / log-green fluorescence (488 laser) and example of gating (red circle) to highly green fluorescent cells . E) application of the SSC-561 scale in linear mode on dot plot for SSC-561/log-green fluorescence showing position of selected cells on E on highly fluorescent and highly granular cells, and highlighting that using log-SSC mode led to miss low SSC-high fluorescent cells (red dotted area).

Supplemental Figures – Figure S6

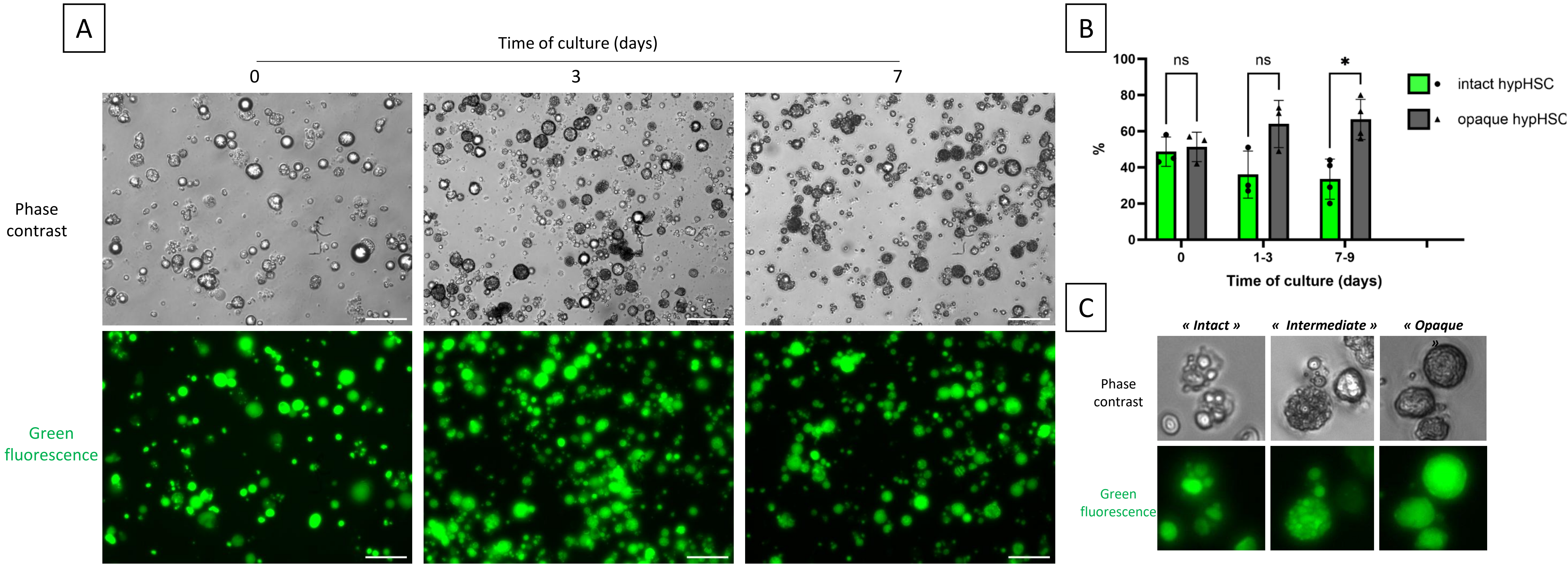

Figure S6 . Evolution of hypHSC phenotype as a function of culture time A) Phase contrast and green fluorescence (λexc=488nm, λem =500-550nm) microscopy images at t=0, 3 and 7 days after sorting. Bars=50µm B) Quantification of opaque and intact hypHSCs by microscopy at a function of time (Mann Whitney test, n=3-4 independent experiments) C) Phase contrast and green fluorescence (λexc=488nm, λem =500-550nm) microscopy images of different phenotypes of hypHSCs according to the state of fluorescent droplets.

Supplemental Figures – Figure S7

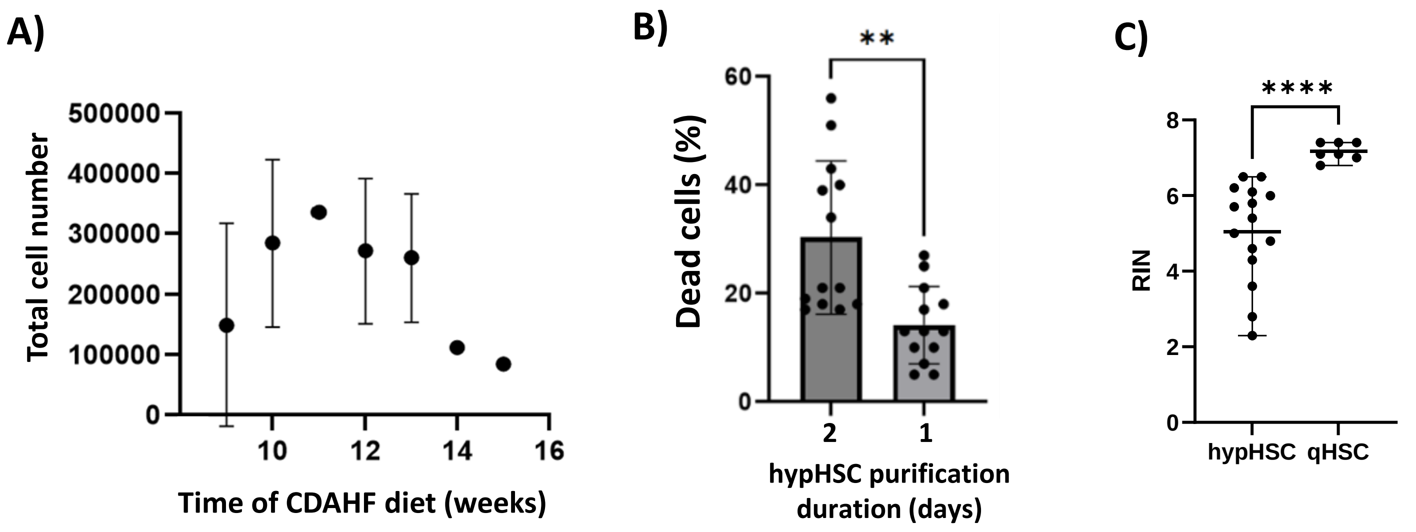

Figure S7 : A) Number of recovered cells after FACS as a function of CDAHFD time. Statistics: for 11 weeks n=1; for 9, 14 and 15 weeks : n=2, for 10 and 13 weeks and for 12 weeks: n=8, mean ± standard deviation, Kruskall-Wallis test not significant. **B) Mortality of hypHSCs collected after sorting according to the duration of purification** (1 or 2 days). Statistics: n = 13 CDAHFD mice for both conditions, mean ± SD, **p<0.005, Mann-Whitney test. **C) RIN values of RNAs extracted from sorted hypHSC and qHSC for RNAseq.** hypHSC n=15; qHSC n=7. ****p<0.0001 Mann-Whitney test

Supplemental Figures – Figure S8

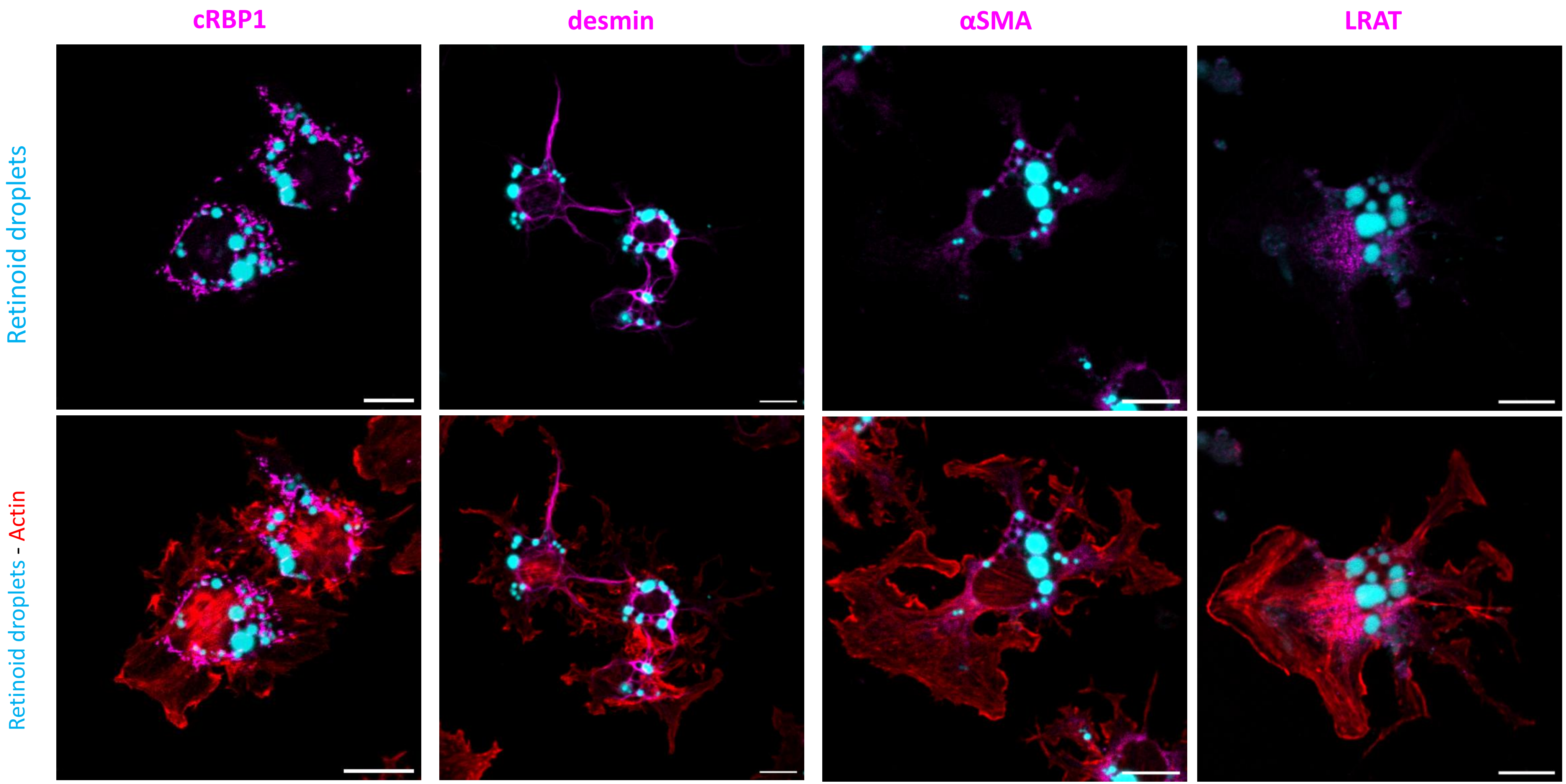

Figure S8. Immunolabeling of stellate cell markers on primary murine HSC purified from SD mouse liver. Purified qHSCs from SD mouse liver were seeded in microwells mounted on a polymer coverslip (Ibidi) at a density ranging from 6000 to 10000 cells/cm² and left to adhere for 16 hours at 37°C / 5% CO2 and fixed with PFA 4% for immunolabeling as described in material and methods. Representative images of cRBP1, desmin, αSMA and LRAT immunostaining (λexc= 638nm, λem= 650-700 nm, magenta) merged with retinoid autofluorescence in the blue range (λexc= 405 nm, λem= 415-490 nm, cyan) (top panels) and actin stained with fluorescent phalloidin, , to visualize cells (λexc= 552 nm, λem= 560-630 nm, red) (bottom panels) acquired by confocal microscopy. Scale bar 10 µm.

Supplemental Figures – Figure S9

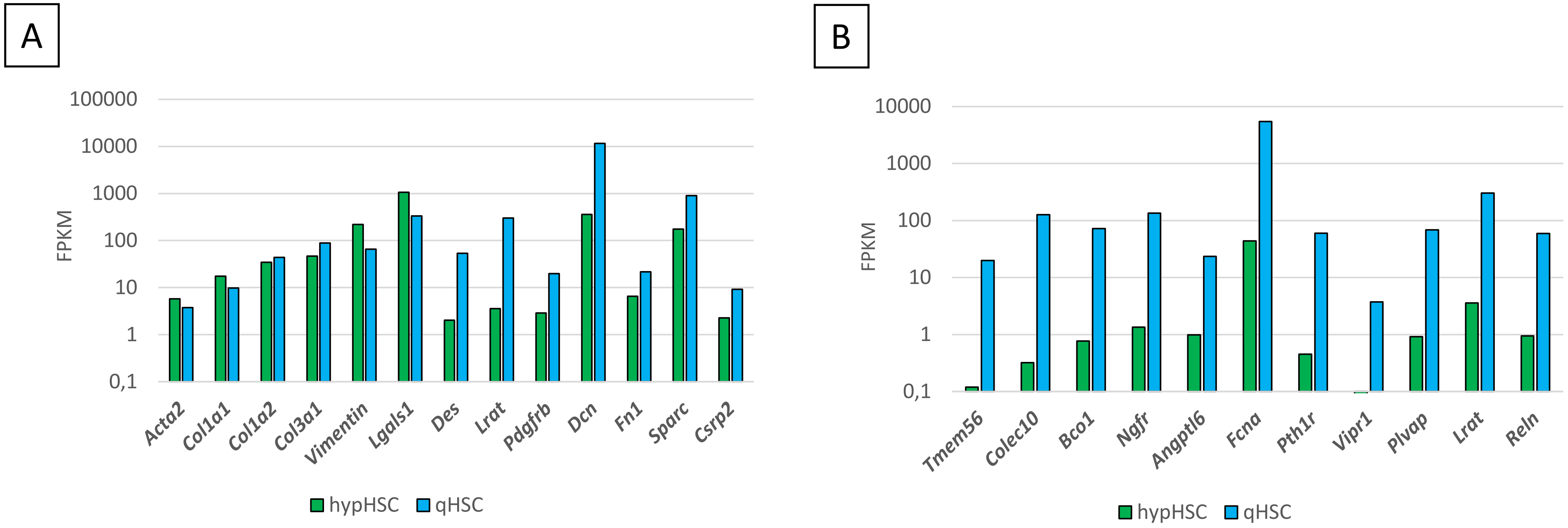

Figure S9. Analysis of the expression of some HSC canonical (A) and specific marker (B) genes by quiescent and hypertrophied HSCs. FPKM (Fragments Per Kilobase of transcription per Million mapped reads) mean of 5 samples for each condition. (A) Acta2= α-SMA; Col1a1, Col1a2 and Col3a1 = collagens 1a1, 1a2 and 3a1; Vim=vimentin; Lgals1 = galectin-1; Des = desmin; Lrat = lecithin retinol acyltransferase*;* Pdgfrb = PDGFR-β; Dcn = decorin; Fn1 = Fibronectin; Sparc = osteonectin; Csrp2 = cysteine- and glycine-rich protein. (B) Tmem56/Tlcd4 (TLC domain containing 4), Colec10 = Collectin subfamily member, Bco1 = betacarotene oxygenase 1, Plvap = plasmalemma vesicle associated protein, Fcna = ficolin a, Pth1r = parathyroid hormone 1 receptor, Angptl6 = angiopoietin like 6, Lrat = lecithin retinol acyltransferase, Reln = Reelin, Vipr1 = Vasoactive intestinal peptide receptor 1, Ngfr = Nerve Growth Factor receptor.

Supplemental Figures – Figure S10

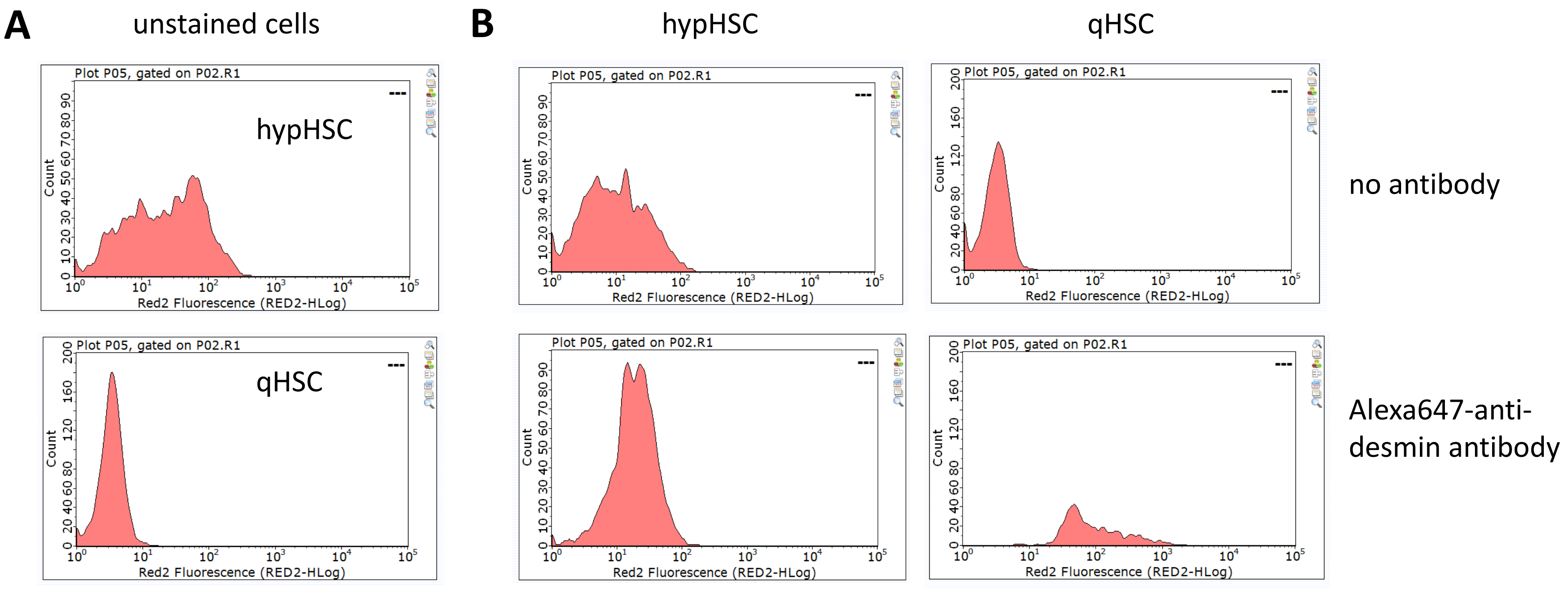

Figure S10. Representative histograms from flow cytometry analysis of sorted hypertrophied and quiescent HSC. Hypertrophied (hypHSC) and quiescent (qHSC) hepatic stellate cells were analyzed by flow cytometry and Red2 fluorescence signals were recorded and displayed as histograms. A) unstained cells. B) unstained cells (up) and cells stained with Alexa647-labeled anti-desmin antibody (down). HypHSC show a very broad fluorescence signal, much more wide-ranging than that of qHSCs. When these two cell types are analyzed by flow cytometry, e.g. with excitation at 647 nm, qHSCs show a classic peak in the low range of fluorescence intensity, whereas hypHSCs show a much wider distribution of cells as a function of fluorescence intensity, with a heterogeneous profile. When both cell types are incubated with an anti-desmin antibody coupled to the Alexa 647 fluorophore, the qHSC peak shifts towards higher intensities, univocally demonstrating labeling of all cells. For hypHSCs, the shape of the distribution profile changes, but the range of intensities remains unchanged, making it impossible to demonstrate specific labeling.

Supplemental Tables - Table S1

gene name	regulation (up/down/NR)	Fold change	P-value	hypHSC		qHSC	
				expression	Rank	expression	Rank
adipor1	up	2.57	6.97E-13	10.57	4033	5.18	7025
sparc	down	3.94	1.53E-04	175.7	440	900.2	58
dcn	down	25.27	4.65E-65	357.15	209	11620.53	2
csrp2	down	3.14	1.16E-05	2.27	9045	9.25	5127
bambi	down	2.67	8.75E-04	0.78	13334	2.74	9305
plin2	up	3.43	4.77E-10	733.77	93	270.2	252
vim	up	4.33	4.52E-15	218.77	338	65.76	1175
pparg	up	90.29	1.30E-13	11.66	3793	0.17	23446
lrat	down	66.45	6.44E-53	3.59	7345	301.73	217
gfap	down	43.48	1.73E-09	0.27	19337	14.17	4013
des	down	20.82	5.58E-14	2.03	9500	53.68	1421
acta2	NR			5.81	8071	3.78	5725
col1a1	NR			17.54	2882	9.86	4963
col1a2	NR			34.41	1795	43.67	1690
col3a1	NR			46.92	1410	88.44	866
notch3	NR			0.07	25761	0.13	24490
timp1	NR			50.73	1327	30.77	2267

Table S1. Regulation of HSC specific genes. Table of HSC specific genes with regulation in hypHSC compared to qHSC, value of fold-change, p-value and corresponding expression value (FPKM) and rank for hypHSC and qHSC. Data normalized using RPKM/FPKM method are used to compare expression between genes within a sample, while fold change between samples (Differential expression analysis) used DEseq2 method of normalization. NR = unregulated, up = up-regulated (pink), down = down-regulated (green).

Supplemental Tables - Table S2

Gene	Name	Regulation	Fold-Change	p-value	qHSC (FPKM)	hypHSC (FPKM)
Lipid uptake and intracellular transport						
Cd36	CD36 molecule	up	489.81	5.80E-28	2.45	938.32
Slc27a1/ Fatp // Fatp1	solute carrier family 27 (fatty acid transporter), member 1	up	10.45	2.22E-10	1.71	14.65
Fabp1	fatty acid binding protein 1, liver	NR	1.35	7.03E-01	681.17	684.77
Fabp5	fatty acid binding protein 5, epidermal	up	379.04	8.33E-57	1.95	574.89
Fabp4	fatty acid binding protein 4, adipocyte	up	301.24	3.36E-50	1.66	387.59
Lpl	lipoprotein lipase	up	13.72	2.99E-45	49.41	542.74
Lipolysis / Lipogenesis						
Gpat2	glycerol-3-phosphate acyltransferase 2, mitochondrial	down	228.81	1.40E-85	127.46	0.44
Nceh1	neutral cholesterol ester hydrolase 1	up	67.75	6.76E-39	0.71	38.05
Pnpla2 (Atgl)	patatin-like phospholipase domain containing 2	up	2.82	2.28E-02	7.52	17.11
Plin2	Perilipin-2	up	3.43	4.77E-10	270.20	733.77
Retinoid metabolism						
Lrat	lecithin-retinol acyltransferase	down	66.45	6.44E-53	301.73	3.59
Dhrs3	dehydrogenase/reductase (SDR family) member 3	up	2.06	3.10E-02	331.14	544.94
Rdh13	retinol dehydrogenase 13 (all-trans and 9-cis)	up	3.14	5.89E-03	10.98	26.63
Rbp1	retinol binding protein 1, cellular	down	11.05	1.80E-20	208.93	14.59

Table S2. Regulation of lipid metabolism pathways in hypHSC *vs* qHSC. Table genes with regulation in hypHSC compared to qHSC, value of fold-change, p-value and corresponding expression value (FPKM) for hypHSC and qHSC. Data normalized using RPKM/FPKM method are used to compare expression between genes within a sample, while fold change between samples (Differential expression analysis) used DEseq2 method of normalization. NR = unregulated, up = up-regulated (pink), down = down-regulated (green).

Supplemental Tables - Table S3

Gene	Sense strand	Antisense stand
**Fn1**	GAGAGGAGTGGGAGCGGTTG	TCCCTTTCCATTCCCGAGGC
**Laminin**	GCCGGGTGAGGAGAACAAAGTA	TGGAGAGGTAGCGGGGAGAAA
**Pdgfra**	AAACAAACGGAGGAGCTGCG	CCCCATAGCTCCTGAGACCTTC
**Pdgfrb**	ACGTGGACCCTGTGCAGTTG	GAGTGCGTCCCAGAACAAGC
**Itgav**	CTTTGGGCTGTGGAATCGCC	AGGATTGCGCTCTTGCCTCT
**IL1b**	TCACAAGCAGAGCACAAGCC	GCATTAGAAACAGTCCAGCCC
**18S**	TTGACGGAAGGGCACCACCAG	GCACCACCACCCACGGAATCG
**PGK1**	GCTGAACTCAAATCTCTGCTG	TCTTTTCCCTTCCCTTCTTCC
**HPRT1**	GCTTACCTCACTGCTTTCC	TTCATCATCGCTAATCACGAC
**MMP12**	GATGAGGCAGAAACGTGGAC	TGGGGTACATTATTGACTTTGGA
**LRAT**	GTCCACAGGCTGAGAAGTTCTAT	AATCCCAAGACAGCCGAAGCA
**Col1a1**	GTC GCT TCA CCT ACA GCA C	CAA TGT CCA AGG GAG CCA C
**Col5a1**	CAA AGG TGA AAA GGG CCA TC	CTC CTT TAG GAC CAG ATG AAC C
**Timp 1**	TCCTAGAGACACACCAGAGCAGA	GGGGAACCCATGAATTTAGCCC
**Timp 2**	GGCTGTGAGTGCAAGATCACT	CACGCGCAAGAACCATCACTT
**aSMA**	GGCTCTGGGCTCTGTAAGG	CTCTTGCTCTGGGCTTCATC
**Vegfa**	TTACTGCTGTACCTCCACC	ACAGGACGGCTTGAAGATG
**TNF**	AGAAAAGCAAGCAGCCAACC	CATAGGCACCGCCTGGAGT
**Thsb2**	CATCGGTGCCAAGCAGTTCC	ACTTGCGGTCCTGCTTCAGT
**Actine**	GCTTCTTTGCAGCTCCTTCG	ACCCATTCCCACCATCACAC

Table S3: Primer sequences used for qPCR analysis of gene expression
